# High-dose chemotherapy and autologous stem cell transplant compared with conventional chemotherapy for consolidation in newly diagnosed primary CNS lymphoma—a randomized phase III trial (MATRix)

**DOI:** 10.1186/s12885-016-2311-4

**Published:** 2016-04-21

**Authors:** Elisabeth Schorb, Juergen Finke, Andrés J. M. Ferreri, Gabriele Ihorst, Kristina Mikesch, Benjamin Kasenda, Kristina Fritsch, Heidi Fricker, Elvira Burger, Olga Grishina, Elke Valk, Emanuele Zucca, Gerald Illerhaus

**Affiliations:** Department of Hematology/Oncology, Freiburg University Medical Center, Freiburg, Germany; Unit of Lymphoid Malignancies, Head Division of OncoHematological Medicine, Department of OncoHematology, San Raffaele Scientific Institute, Milan, Italy; Clinical Trials Unit, Freiburg University Medical Center, Freiburg, Germany; Clinic of Hematology, Oncology and Palliative Care, Klinikum Stuttgart, Kriegsbergstr.60, Stuttgart, 70174 Germany; Oncology Institute of Southern Switzerland, San Giovanni Hospital, Bellinzona, Switzerland

**Keywords:** Primary central nervous system lymphoma (PCNSL), High-dose chemotherapy (HDT), Autologous stem cell transplantation (ASCT), Conventional chemotherapy, Randomized controlled trial

## Abstract

**Background:**

Primary central nervous system lymphoma (PCNSL) is a highly aggressive Non-Hodgkin lymphoma (NHL) with rising incidence over the past 30 years in immunocompetent patients. Although outcomes have improved, PCNSL is still associated with inferior prognosis compared to systemic NHL. Many questions regarding the optimal therapeutic approach remain unanswered.

**Methods/Design:**

This is a randomized, open-label, international phase III trial with two parallel arms. We will recruit 250 patients with newly diagnosed PCNSL from approximately 35 centers within the networks of the German Cooperative PCNSL study group and the International Extranodal Lymphoma Study Group. All enrolled patients will undergo induction chemotherapy consisting of 4 cycles of rituximab 375 mg/m^2^/d (days 0 & 5), methotrexate 3.5 g/m^2^ (d1), cytarabine 2 × 2 g/m^2^/d (d2-3), and thiotepa 30 mg/m^2^ (d4) every 21 days. All patients will undergo stem-cell harvest after the second cycle. After 4 cycles of induction chemotherapy, patients achieving partial or complete response will be centrally randomized to 2 different consolidation treatments: (A) conventional-dose immuno chemotherapy with rituximab 375 mg/m^2^ (d0), dexamethasone 40 mg/d (d1-3), etoposide 100 mg/m^2^/d (d1-3), ifosfamide 1500 mg/m^2^/d (d1-3) and carboplatin 300 mg/m^2^ (d1) (R-DeVIC) or (B) high-dose chemotherapy with BCNU (or busulfan) and thiotepa followed by autologous stem cell transplantation (HCT-ASCT). The objective is to demonstrate superiority of HCT-ASCT compared to R-DeVIC with respect to progression-free survival (PFS, primary endpoint). Secondary endpoints include overall survival (OS), treatment response and treatment-related morbidities. Minimal follow-up after treatment completion is 24 months.

**Discussion:**

The rationale for consolidation treatment in PCNSL is to eliminate residual lymphoma cells and to decrease the risk for relapse. This can be achieved by agents crossing the blood brain barrier either applied at conventional doses or at high doses requiring autologous stem cell support. HCT-ASCT has been shown to be feasible and highly effective in patients with newly-diagnosed PCNSL. However, it is unclear whether HCT-ASCT is really superior compared to conventional-dose chemotherapy after an intensified antimetabolites-based immunochemotherapy in patients with newly-diagnosed PCNSL. To answer this question, we designed this investigator initiated randomized phase III trial.

**Trial registration:**

German clinical trials registry DRKS00005503 registered 22 April 2014 and ClinicalTrials.gov NCT02531841 registered 24 August 2015.

**Electronic supplementary material:**

The online version of this article (doi:10.1186/s12885-016-2311-4) contains supplementary material, which is available to authorized users.

## Background

Primary CNS lymphoma (PCNSL) accounts for 1 to 2 % of all Non-Hodgkin’s lymphomas (NHL) and for 2 to 7 % of all primary CNS tumors. Its incidence has increased over the past 30 years.

Prognosis without treatment resembles that of systemic high-grade NHL, with a median survival of approximately 3 months. Although therapy has improved the outcome of patients with PCNSL, prospective clinical trials are rare compared to systemic NHL and many questions regarding the optimal therapeutic approach remain unanswered. Currently, high-dose methotrexate (HD-MTX) is considered the most efficient known cytostatic agent for PCNSL [1, 2]. Several drugs have been combined with HD-MTX to improve outcome, but only one randomized trial demonstrated superiority of additional high-dose cytarabin (HD-AraC) compared to single agent HD-MTX treatment so far [3]. Other agents such as lomustine, procarbazine, vinca alkaloids, temozolomide and thiotepa have also been added to HD-MTX, revealing promising remission rates and acceptable toxicity profiles. A single-arm phase II trial assessing the chemotherapy combination named “MATILDE” has included thiotepa [4], resulting in an overall response rate (ORR) of 72 % and a complete remission rate (CRR) of 46 %, with a 5-year survival of 42 %. The use of thiotepa is justified by its excellent bioavailability in the CNS and its high efficacy in aggressive lymphoma and in reported trials on PCNSL [4–6]. Rituximab in addition to systemic chemotherapy is the current standard for treating systemic B-cell lymphomas [7]. First results of the randomized phase II International Extranodal Lymphoma Study Group (IELSG) 32 trial show a significant improvement of response, failure free survival and overall survival by the addition of rituximab to standard HD-MTX/HD-AraC. Further addition of thiotepa to HD-MTX/HD-AraC shows even further improvement, but not reaching statistical significance [8].

Recent trials have demonstrated that consolidating strategies with non-cross-resistant cytostatic agents in first-line therapy yield promising results in treating PCNSL [9]. Another regimen with dexamethasone, etoposide, ifosfamide and carboplatin (DeVIC) has been applied in recurrent/refractory and in newly-diagnosed PCNSL with good results. A retrospective analysis of 21 patients with newly-diagnosed PCNSL who received DeVIC chemotherapy followed by whole brain radiation therapy (WBRT) showed high efficacy with ORR to chemotherapy (DeVIC) of 95 % in newly-diagnosed PCNSL and 83 % in refractory and recurrent PCNSL [10].

Based on experience in other hematological malignancies, such as relapsed systemic diffuse large B-cell lymphoma, and the need for effective consolidation treatment, high-dose chemotherapy supported by autologous stem cell transplantation (HDT-ASCT) has also been investigated in PCNSL. The rationale for HDT-ASCT in PCNSL is to achieve therapeutic drug concentration in the CNS tissues and sanctuaries, and to overcome chemo resistance [11, 12]. In recent trials, we demonstrated a high rate of long-lasting remissions in PCNSL patients treated with HDT-ASCT, with or without WBRT [5, 6]. In a pilot and phase-II study, we treated 30 patients with PCNSL ≤65 years with sequential induction chemotherapy including three cycles of HD-MTX, HD-AraC, and thiotepa followed by stem-cell harvest. The conditioning regimen consisted of carmustine and thiotepa followed by ASCT; WBRT was given as consolidation [5]. Twenty-three of the 30 patients proceeded to HDT-ASCT resulting in complete remission (CR) and partial remission (PR) in 15 and 8 patients, respectively. With a median follow-up of 63 months, the 5-year OS was 69 % for all patients and 87 % for those completing HDT-ASCT. In a further trial, induction chemotherapy has been intensified, thiotepa dose was doubled, and only those patients not achieving CR after induction therapy underwent WBRT (“Freiburg Protocol”) [6]. Seven of 11 patients were in CR following ASCT, and three in PR underwent post-ASCT radiotherapy. After a median follow-up of 25 months, 3-year OS was 77 %. None of the patients suffered from severe neurotoxicity during the follow-up period. Both trials have suggested a curative potential of HDT-ASCT in young PCNSL patients. This concept, supplemented by rituximab immunotherapy, was evaluated in a phase II trial (ClinicalTrials.gov Identifier: NCT00647049). Preliminary results revealed an ORR of 91 % (77 % CR and 14 % PR) for patients treated with HDT-ASCT (*n* = 73) [13]. After a median follow-up of 35 months, the 3-year OS was 77.6 % for all patients and 87.1 % for patients after HDT-ASCT. In light of these findings, we initiated an ongoing international randomized phase-II trial in collaboration with the IELSG. This trial has 2 randomizations: in the 1^st^ randomization patients are allocated to primary chemotherapy with HD-MTX and HD-Ara-C with or without thiotepa, and with or without rituximab. The second randomization allocates to consolidation therapy with WBRT vs. HDT-ASCT (ClinicalTrials.gov Identifier: NCT01011920). In this trial, we aim to determine the best induction treatment as well as the superiority of HDT-ASCT or WBRT (the current standard for consolidation after HD-MTX-based systemic treatment) as consolidation treatment. First results of the randomized phase II IELS32 trial show that the addition of thiotepa and rituximab to MTX and Ara-C is associated with significantly improved CRR and ORR [8]. The efficacy of HDT-ASCT has been shown in several phase II trials in PCNSL patients, both as upfront and salvage therapy, and has yielded promising results concerning response and survival rates [5, 6, 14–16]. However, as there have been no randomized trials demonstrating a benefit of this concept over conventional optimized combination chemotherapy, we designed the MATRix trial to determine whether HDT-ASCT is superior to conventional-dose chemoimmunotherapy (R-DeVIC) as consolidation after intensified induction treatment in patients with newly-diagnosed PCNSL.

## Methods

### Study design

This is a randomized, controlled, open-label, international phase III trial with 2 parallel arms comparing HDT-ASCT with conventional chemotherapy for consolidation in newly diagnosed primary CNS lymphoma. Patients will be recruited from 35 centers in Germany. Furthermore the International Extranodal Lymphoma Study Group (IELSG) will participate in the study and recruit patients. Approval and permission to conduct the study was obtained from all participating centers. The study protocol was approved by the leading ethics committee (Ethik-Kommission Landesärztekammer Baden Württemberg) and the local ethics committees. A complete list of the committees that approved the study is given in the Supplementary Material (see Additional file 1: Table S1). The protocol was also subject to authorization by the competent authorities as mandatory by federal law. All participants have to provide written informed consent. The trial was assigned the EudraCT number 2012-000620-17 and is registered at German clinical trials registry (DRKS00005503, registration date 22 April 2014) and ClinicalTrials.gov (NCT02531841, registration date 24 August 2015). The SPIRIT checklist of the trial is given in the Supplemental Material (see Additional file 2).

### Study objectives and endpoints

The primary objective of the MATRix trial is to demonstrate superior efficacy of HDT-ASCT compared to conventional chemotherapy. The primary endpoint of this study is PFS, defined as time from the date of randomization to the date of lymphoma progression, relapse or death from any cause with possible censoring at the date of last visit of follow-up.

Secondary endpoints include complete remission rate (CRR) on day 60 after randomization; duration of response (time from CR, unconfirmed CR or PR until relapse, death or last follow-up visit); OS; and quality of life (QOL, according to EORTC QLQ-C30). Secondary safety endpoints are (serious) adverse events, toxicity (according to NCI-CTCAE v.4.0) and neurotoxicity (according to Mini-Mental State Examination (MMSE), EORTC QLQ-BN20 and neuro-psychological battery).

### Eligibility criteria

Immunocompetent patients with newly-diagnosed PCNSL of B-cell immunophenotype, aged 18–65 years with an ECOG Performance Status ≤3, or 66–70 years with an ECOG Performance Status ≤2 are eligible. Randomization is limited to patients demonstrating a successful stem cell harvest, response to treatment, and confirmation of diagnosis by the central pathological review. For further details on inclusion and exclusion criteria please see Table 1.

**Table 1:** Inclusion and exclusion criteria

Inclusion criteria	1.Immunocompetent patients with newly-diagnosed primary central nervous system B-cell lymphoma 2.Age 18–65 years irrespective of ECOG or 66–70 years (with ECOG Performance Status ≤2) 3.Histologically or cytologically assessed diagnosis of B-cell lymphoma by local pathologist. 4.Diagnostic sample obtained by stereotactic or surgical biopsy, CSF cytology examination or vitrectomy 5.Disease exclusively located in the CNS 6.At least one measurable lesion 7.Previously untreated patients (previous or ongoing steroid treatment admitted) 8.Sexually active patients of childbearing potential who agree to take adequate contraceptive measures during study participation 9.Written informed consent obtained according to international guidelines and local laws by patient or authorized legal representative in case patient is temporarily legally not competent due to his or her disease
Additional randomization criteria	1.Sufficient stem cell harvest (≥ 5 x 106 CD34+ cells/kg of body weight) 2.Complete remission, unconfirmed complete remission or partial remission 3.Central pathology results confirming local results
Exclusion criteria	1.Congenital or acquired immunodeficiency 2.Systemic lymphoma manifestation (outside the CNS) 3.Isolated ocular lymphoma without manifestation in the brain parenchyma or spinal cord 4.Previous or concurrent malignancies with the exception of surgically cured carcinoma insitu of the cervix, carcinoma of the skin or other kinds of cancer without evidence of disease for at least 5 years 5.Previous Non-Hodgkin lymphoma at any time 6.Inadequate bone marrow (platelet count decreased ≥ CTC grade 1, anemia ≥ CTC grade 1, neutrophil count decreased ≥ CTC grade 1), renal (creatinine clearance <60 ml/min), cardiac (ejection fraction decreased ≥ CTC grade 2), or hepatic function (blood bilirubin increased ≥ CTC grade 2, alanine aminotransferase increased ≥ CTC grade 2, aspartate aminotransferase increased ≥ CTC grade 2 or gamma-GT increased ≥ CTC grade 2) 7.HBsAg, anti-HBc or HCV positivity 8.HIV infection, previous organ transplantation or other clinical evident form of immunodeficiency 9.Concurrent treatment with other experimental drugs or participation in a clinical trial within the last thirty days before the start of this study 10.Symptomatic coronary artery disease, cardiac arrhythmias uncontrolled with medication or myocardial infarction within the last 6 months (New York Heart Association Class III or IV heart disease) 11.Severe non-compensated pulmonary disease (IVC <55 %, DLCO <40 %) 12.Third space fluid accumulation >500 ml 13.Hypersensitivity to study treatment or any component of the formulation 14.Taking any medications likely to cause interactions with the study medication 15.Known or persistent abuse of medication, drugs or alcohol 16.Patient without legal capacity and who is unable to understand the nature, significance and consequences of the study and without designated legal representative 17.Persons who are in a relationship of dependency/employment to the sponsor and/ or investigator 18.Any familial, sociological or geographical condition potentially hampering compliance with the study protocol and follow-up schedule 19.Concurrent (or planned) pregnancy or lactation 20.Fertile patients refusing to use safe contraceptive methods during the study

*ECOG* Eastern Cooperative Oncology Group Performance Status, *CSF* cerebrospinal fluid, *CNS* central nervous system, *CTC* common toxicity criteria, *HBsAg* hepatitis B surface antigen, anti-*HBc* hepatitis B core antigen antibody, *HCV* hepatitis C virus, *HIV* human immunodeficiency virus, *IVC* inspiratory vital capacity, *DLCO* diffusing capacity of the lung for carbon monoxide

### Randomization methodology

A randomized design (block randomization with randomly-varying block sizes with an allocation ratio of 1:1) is applied in order to ensure comparability of the treatment groups. Central randomization by fax will be performed to guarantee concealment of the treatment allocation. Stratification according to response status (CR or PR) after 4 courses of induction chemotherapy will be performed. Patients with SD or PD after induction treatment will be treated off-study. No stratification by study centers will take place, because many centers having small numbers of patients will be included in the trial. Randomization will take place after 4 cycles of induction therapy, i.e. immediately before starting treatment with either HDT-ASCT or R-DeVIC in order to enable an analysis according to the intention-to-treat (ITT) principle with as few protocol violators or drop-outs as possible. The block lengths will be documented separately and will not be disclosed to the centers. The randomization code will be produced by validated programs based on the Statistical Analysis System (SAS®).

### Treatment schedule

**Fig. 1:**
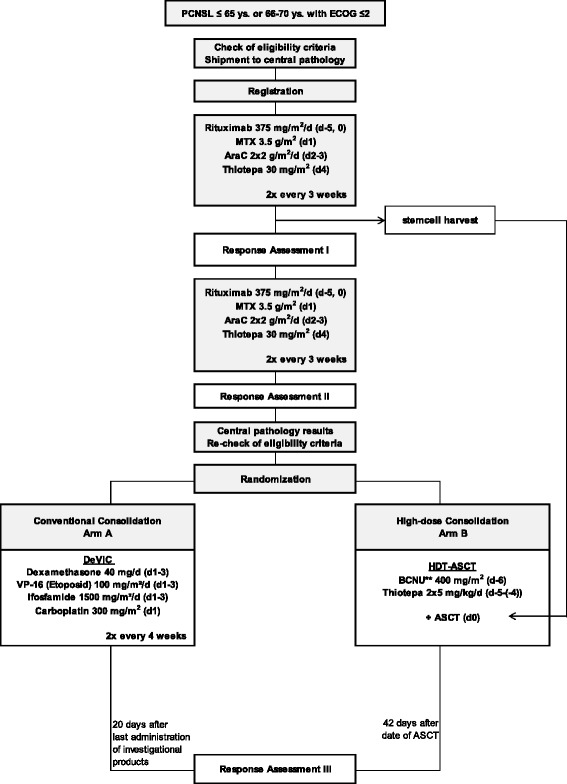
Intervention Scheme. Patients with PD after two cycles of induction treatment, PD or SD after four cycles, insufficient bone marrow recorvery after chemotherapy or insufficient stem cell harvest are not eligible for randomization; patients with complete remission, unconfirmed complete remission or partial remission after completion of therapy will undergo regular follow up; patients with stable disease or progressive disease after completion of therapy will undergo salvage treatment according to investigator’s choice. PCNSL = primary central nervous system lymphoma; ECOG = Eastern Cooperative Oncology Group Performance Status; d = day; MTX = methotrexate; AraC = cytarabine; HDT-ASCT = high-dose chemotherapy followed by autologous stem cell transplantation; BCNU = carmustine,** if BCNU is not available at study site, busulfan can be used instead; PD = progressive disease; SD = stable disease; PR = partial remission; CR = complete remission; uCR = unconfirmed complete remission

The treatment schedule is summarized in Fig. 1 (Intervention Scheme). Randomization to either the conventional consolidation (arm A) or HCT-ASCT consolidation (arm B) will take place after 4 cycles of induction therapy after re-checking inclusion/exclusion criteria and checking the randomization criteria.

### Induction treatment

Induction treatment consists of four cycles (every 3 weeks) of induction chemotherapy similar to the MATRix regimen [8]. Rituximab will be given intravenously at 375 mg/m^2^ on day 0 and 5 of each cycle. High-dose MTX will be administered on day 1 of each induction treatment cycle intravenously at 0.5 g/m^2^ in 15 min and then 3 g/m^2^ as a 3-h infusion. Cytarabine will be given intravenously at 2 g/m^2^ over 1 h, twice a day (every 12 h) on two consecutive days (days 2 and 3). Thiotepa will be given intravenously at 30 mg/m^2^ over 30 min on day 4 during induction chemotherapy. Stem-cell harvest will be performed after the second cycle. The objective is to harvest a minimum of 5 × 10^6^ CD34+ cells/kg of body weight with as few as possible leukapheresis sessions on consecutive days. CD34+ cells are to be collected, processed and stored according to conventional guidelines.

### Consolidation treatment

Patients assigned to arm A will receive two cycles (every 3 weeks) of conventional consolidation therapy according to the R-DeVIC protocol: Rituximab 375 mg/m^2^ over 1.5 h on day 0; dexamethasone given intravenously at a dose of 40 mg over 15 min (d1-3); etoposide 100 mg/m^2^ over 2 h (d1-3); ifosfamide 1500 mg/m^2^ over 2 h (d1-3) and carboplatin 300 mg/m^2^ over 1 h (d1).

Patients assigned to arm B will receive HCT-ASCT with carmustine (BCNU) and thiotepa: BCNU 400 mg/m^2^ over 1 h on day −6, thiotepa 5 mg/kg over 2 h twice a day (every 12 h) on two consecutive days (days −5 and −4). If BCNU is not available at the investigational site, busulfan (3.2 mg/kg over 2 h on two consecutive days (days −8 and −7). Autologous stem cell reinfusion will be performed according to standard procedures on day 0.

### Assessments and follow up

At each visit, the following parameters will be evaluated: Eastern Cooperative Oncology Group (ECOG) Performance Status, vital signs, physical and a neurological examination, laboratory profile, and adverse events, which will be graded according to NCI-CTCAE v.4.0.

Tumor response will be assessed following the IPCG response criteria [17]. Response assessment by brain MRI will be done after the second and fourth course and on day 60 after randomization. After the end of treatment, disease status will be assessed every 3 months during the first 2 years, every 6 months during the following 3 years, and annually thereafter. The trial flow chart indicating the assessments during the trial and the follow up period is given as appendix of the SPIRIT checklist in the Supplemental Material (see Additional file 2).

Tumor size, location(s) (only at screening and in case of PD) and manifestation (singular/multiple; only at screening) will be evaluated at the aforementioned time points. In case of multiple tumors, one reference tumor will be measured, the response being evaluated by comparison to the screening MRI. Response evaluation during the trial will be determined by an independent radiological review committee not involved in the study design and blinded to treatment assignment.

### Sample size estimation

Sample size calculation is based on the primary endpoint PFS. We are assuming that the PFS rate for patients treated with the conventional intervention (arm A, R-DeVIC) is approximately 50 % after 3 years. To compare the two treatment groups, a hazard ratio of 1.8 of the conventional intervention (R-DeVIC) versus the HCT-ASCT (arm B) is considered clinically relevant. This corresponds to a PFS rate after 3 years of 68 % in the HCT-ASCT group (arm B). To detect a difference between arms A and B with a power of 80 % at a two-sided significance level of 5 % under this assumption 92 PFS defining events are required. Assuming an exponential model for survival, an accrual period of 3 years and an additional follow-up time of 2 years, we will need to enroll at least 200 patients. With an expected drop-out of 10 %, 220 patients will be randomized. Furthermore, we anticipate that some patients (about 10 %) will fail to achieve complete or partial remission during the first 4 chemotherapy cycles and will not be randomized. We therefore assume we will have to include approximately 250 patients in the study (start induction treatment).

### Statistical analysis

The primary analysis will be conducted according to the intention-to-treat principle and will therefore be based on the full analysis set (FAS). The FAS includes all randomized patients in whom therapy after randomization was started, and patients are analyzed as belonging to their randomized arm, regardless of whether they refused or discontinued therapy, or whether other protocol deviations are known. Patients will be censored at the time of last follow-up provided no event of interest has occurred so that as many patients as possible can be included in the analysis.

The primary endpoint PFS will be analyzed with a multivariable Cox proportional hazards model, containing the randomized treatment as explanatory variable adjusted for the stratification variable response status. The test of the primary hypothesis (null hypothesis: equality of PFS rates) will be conducted within this model. The treatment effect will be described by the estimated hazard ratio from this model and will be presented with a two-sided 95 % confidence interval. The null hypothesis will be rejected if the value 1 is not contained in the two-sided 95 % confidence interval for the hazard ratio describing the relation between treatment groups. Additionally, the PFS rates will be estimated by the Kaplan-Meier method.

The endpoint OS will be analyzed in the same way as described for PFS. The endpoint CR rate will be analyzed as the dependent variable of a logistic regression model with treatment assignment as independent variable. The endpoint response duration will be estimated with a Cox-regression model. Death without former progression will be analyzed as competing event. The regression models allow the inclusion of further potentially important prognostic factors. Details will be determined in a Statistical Analysis Plan (SAP) to be finalized before the analysis starts. For the evaluation of CR on day 60 after randomization, patients not completing therapy will be counted as non-responders. With respect to the endpoint QOL, the treatment groups will be compared descriptively according to the EORTC manual. All p-values from analyses of secondary endpoints will be interpreted in a descriptive sense.

Further descriptive analyses will consider all patients included in the trial, i.e. data from start of induction therapy with Rituximab, HD-MTX, HD-Ara-C, and thiotepa will be analyzed. We will analyze PFS and OS starting at registration for all patients, and we will consider three groups (two treatment arms, not randomized).

### Subgroup analyses

Subgroup analyses will be conducted based on response status (CR versus PR) as evaluated by contrast enhanced MRI at the time of randomization. Of note, response status is a stratification factor, thus the number of patients with either CR or PR between the two arms will very likely be balanced.

We hypothesize that particularly those patients with PR will benefit more from the more aggressive approach HDT-ASCT than patients with CR, because patients who do not respond completely after 4 cycles of conventional chemotherapy might be suffering from a lymphoma that is more resistant to conventional chemotherapy than the lymphomas in those patients who had already achieved a complete response. Therefore, the response status can be considered as a surrogate for a certain lymphoma biology that tends to be more aggressive, something not yet well understood.

We will estimate these subgroup effects for PFS and OS using Cox regression analyses including the following variables in the models: treatment allocation and response status at randomization. An appropriate interaction term (treatment allocation*response status) will be added to the model. We will illustrate subgroup effects using forest plots and provide the P value for the interaction test. Whether the number of events (around 7 per variable) suffices to designate OS will have to be seen. If not, we will conduct the subgroup analyses only for PFS. Based on the IELSG risk score, [18] we will evaluate the prognostic impact (PFS and OS) of the following baseline variables at the time of study inclusion before starting any chemotherapy: age (as continuous variable), performance score (ECOG 0–1 versus ECOG >1), liquor-protein elevation (yes versus no), involvement of deep-brain structures (yes versus no), and elevated serum LDH (yes versus no). We will use Cox regression analyses without variable selection procedures to explore these risk factors.

### Quality assurance and safety

Our study data will be managed using the DAMAST Version 9.2, a proprietary data management system based on the software package SAS®, which has been developed, validated and is maintained by the Clinical Trials Unit Freiburg (CTU). Double data entry will be performed by two different persons (with the exception of free text). SAS® software will be used to review the data for completeness, consistency and plausibility.

### Data Monitoring Committee

An independent Data Monitoring Committee (DMC) will be established. The DMC will consist of two medical scientists and one statistician with longstanding experience in clinical trials. The DMC’s function is to monitor the study’s course and if necessary make recommendations to the steering committee for study discontinuation, modification or continuation. The underlying principles for the DMC are the patients' ethical and safety aspects. It is the task of the DMC to examine whether the study’s conduct is still ethically justifiable, whether security of the patients is ensured and whether the study’s conduct is acceptable. The DMC will be informed about adherence to the protocol, patient recruitment, observed serious adverse events and deaths by receiving the development safety update reports (DSURs) at regular intervals. Recommendations on further continuation or modification of the study will be given to the steering committee. The composition and responsibilities of the DMC, the structure and procedures of its meetings, and its relationship to other key study team members (steering committee), will be laid down in a separate DMC charter.

## Discussion

Untreated PCNSL has a dismal prognosis; its median survival time is approximately 3 months. Current treatment strategies have improved survival and shown curative potential in a considerable number of patients. Similar to other hematological diseases, the rationale for consolidation in PCNSL is to eliminate minimal residual disease. There is evidence that HDT-ASCT with carmustine or busulfan and thiotepa is feasible and highly effective both in patients with newly-diagnosed and relapsed PCNSL [5, 6, 14].

The rationale behind the impact of HDT-ASCT in PCNSL is the delivery of blood brain barrier (BBB) penetrating agents into the CNS at several-fold higher concentrations than conventional therapy [11, 12]. The efficacy of HDT-ASCT has been investigated in several phase II trials for primary, relapsed, or refractory PCNSL, revealing promising response and survival rates [5, 6, 15, 16]. However, although urgently needed, there have been no randomized trials demonstrating this concept’s superiority to optimized, conventional-dose polychemotherapy. The question we now aim to answer is whether HDT-ASCT is superior to conventional therapy as consolidation after intensified immunochemotherapy in patients with newly-diagnosed PCNSL. Quality assurance will be acquired by independent radiological and pathologic review committees not involved in the study’s conception as well as by a blinded data review undertaken after the end of the recruitment period and the planned follow-up period. Therefore our study follows high-level methodological standards.

We have been chosen the DeVIC combination chemotherapy regimen for conventional consolidation, because its components cross the BBB and consist of multidrug resistant-unrelated agents. HD-MTX-based induction chemotherapy has been optimized over the past years. An international randomized phase-II-trial was recently conducted in collaboration with the IELSG to determine both the best induction treatment and the superiority of HDT-ASCT over WBRT as consolidation treatment. Apart from the expected hematological toxicity, the preliminary data have revealed good tolerability and efficacy of the combination of rituximab, HD-MTX, HD-Ara-C and thiotepa (MATRIX regimen) as induction regimen [8]. In comparison to the IELSG32 trial, the induction treatment schedule was modified by administering rituximab on day 0 and +5. We assume that the application of two doses of rituximab—one before and one after chemotherapy - results in a superior intracerebral concentration compared to bi-weekly dosing.

Results of the MATRix trial are expected to provide high-level evidence with regard to the best consolidation treatment in newly diagnosed PCNSL.

## Additional files

Additional file 1: Table S1. List of ethics committees. (PDF 32 kb) Additional file 2: SPIRIT checklist. (PDF 552 kb)

## Abbreviations

Anti-HBc: hepatitis B core antigen antibody
ASCT: autologous stem cell transplantation
BBB: blood brain barrier
BCNU: carmustine
BfArM: Bundesinstitut für Arzeimittel und Medizinprodukte
CR: complete remission
CSF: cerebrospinal fluid
CTU: Clinical Trials Unit
d: day
DLBCL: diffuse large B-cell lymphoma
DLCO: diffusing capacity of the lung for carbon monoxide
ECOG: Eastern Cooperative Oncology Group
EORTC QLQ-C30: European Organisation for Research and Treatment of Cancer Questionnaire Core-30
HBsAg: hepatitis B surface antigen
HCV: hepatitis C virus
HD-AraC: high-dose cytarabine
HD-MTX: high-dose methotrexate
HDT: high-dose chemotherapy
HIV: human immunodeficiency virus
IELSG: International Extranodal Lymphoma Study Group
IVC: inspiratory vital capacity
MMSE: Mini-Mental State Examination
NCI-CTCAE: National Cancer Institute - Common toxicity criteria
NHL: Non Hodgkin Lymphoma
ORR: overall response rate
OS: overall survival
PCNSL: primary central nervous system lymphoma
PD: progressive disease
PFS: progression free survival
PR: partial remission
QoL: quality of life
R-DeVIC: dexamethasone, etoposide, ifosfamide, carboplatin
RT: radiotherapy
(S)AE: (serious) adverse event
SAP: statistical analysis plan
SAS: Statstical Analysis System
SD: stable disease
uCR: unconfirmed complete remission
