# Supplementary material for: High-dose chemotherapy and autologous stem cell transplant compared with conventional chemotherapy for consolidation in newly diagnosed primary CNS lymphoma—a randomized phase III trial (MATRix)
Source: BMC Cancer. 2016 Apr 21;16:282. doi: 10.1186/s12885-016-2311-4 (PMC4839072; doi:10.1186/s12885-016-2311-4)
Supplement: Additional file 1: Table S1. — List of ethics committees. (PDF 32 kb) [file 12885_2016_2311_MOESM1_ESM.pdf]

MATRix / IELSG43 Studie:
EudraCT-Nummer: 2012-000620-17

Participating centers und ethics committees

	Leading center	Principal Trial Coordinator	Leading ethics committee
1	**Klinikum Stuttgart<br>Klinik für Hämatologie,<br>Onkologie und Palliativmedizin<br>Stuttgart Cancer Center /<br>Tumorzentrum Eva Mayr-Stihl<br>Kriegsbergstr. 60<br>70174 Stuttgart**	**LKP:<br>Prof. Dr. Gerald Illerhaus<br>Stellvertreterin:<br>Dr. Kristina Mikesch**	**Ethik-Kommission bei der<br>Landesärztekammer Baden-<br>Württemberg<br>Jahnstraße 40<br>70597 Stuttgart**
	**Participating centers**	**PI & Deputy PI:**	**Local ethics committees:**
2	Universitätsklinikum Aachen<br>Klinik für Onkologie, Hämatologie<br>und Stammzelltransplantation<br>(Medizinische Klinik IV)<br>Pauwelsstr. 30<br>52074 Aachen	Prüfer:<br>Dr. med. Jens Panse<br>Stellvertreterin:<br>Dr. med. Mareike Tometten	Ethik-Kommission an der<br>Medizinischen Fakultät<br>der RWTH Aachen<br>Pauwelsstraße 30<br>52074 Aachen
3	Klinikum Augsburg<br>II. Medizinische Klinik<br>Cancer Center Augsburg<br>Stenglinstr.2<br>86156 Augsburg	Prüfer:<br>Prof. Dr. Martin Trepel<br>Stellvertreter:<br>PD Dr. Andreas Rank	Ethik-Kommission der Bayerischen<br>Landesärztekammer<br>Mühlbaurstraße 16<br>81677 München
4	Städtisches Klinikum<br>Braunschweig gGmbH<br>Medizinische Klinik III<br>Hämatologie und Onkologie<br>Celler Straße 38<br>38114 Braunschweig	Prüfer:<br>Dr. Sebastian Schroll<br>Stellvertreter:<br>Dr. Heiko Hütten	Ethik-Kommission bei der<br>Ärztekammer Niedersachsen<br>Berliner Allee 20<br>30175 Hannover
5	Klinikum Bremen-Mitte gGmbH<br>Medizinische Klinik I<br>St.Jürgen.Str. 1<br>28177 Bremen	Prüfer:<br>Prof. Dr. Bernd Hertenstein<br>Stellvertreter:<br>Dr. Stefan Kaun	Ethik-Kommission des Landes Bremen<br>Institut für klinische Pharmakologie<br>Klinikum Bremen-Mitte gGmbH<br>St.-Jürgen-Straße 1<br>28177 Bremen
6	Klinikum Chemnitz gGmbH<br>Klinik für Innere Medizin III<br>Bürgerstraße 2<br>09113 Chemnitz	Prüfer:<br>PD Dr. Mathias Hänel<br>Stellvertreterin:<br>Dr. Anke Morgner	Ethik-Kommission der sächsischen<br>Landesärztekammer<br>Schützenhöhe 16<br>01099 Dresden
7	Universitätsklinikum Düsseldorf<br>Klinik für Hämatologie, Onkologie<br>und Klinische Immunologie<br>Moorenstr. 5<br>40225 Düsseldorf	Prüfer:<br>Prof. Dr. Guido Kobbe<br>Stellvertreter:<br>Dr. Mustafa Kondakci	Ethik-Kommission der Medizinischen<br>Fakultät der Heinrich-Heine-Universität<br>Düsseldorf<br>Kinderklinik, Geb. 13.41<br>Moorenstr. 5<br>40225 Düsseldorf
8	Universitätsklinikum Erlangen<br>Medizinische Klinik 5<br>Hämatologie und Internistische<br>Onkologie<br>Ulmenweg 18<br>91054 Erlangen	Prüfer:<br>Prof. Dr. Stephan Krause<br>Stellvertreter:<br>Dr. Bernd Spriewald	Ethik-Kommission der Medizinischen<br>Fakultät<br>Friedrich-Alexander-Universität<br>Erlangen-Nürnberg<br>Krankenhausstraße 12<br>91054 Erlangen
9	Universitätsklinikum Essen<br>Klinik für Hämatologie<br>Hufelandstraße 55<br>45122 Essen	Prüfer:<br>PD Dr. Alexander Röth<br>Stellvertreter:<br>PD Dr. Andreas Hüttmann	Ethik-Kommission der Med. Fakultät<br>der Universität Duisburg-Essen<br>Jakobs Universitätsklinikum Essen<br>Robert-Koch-Str. 9-11<br>45122 Essen

Participating centers und ethics committees

10	Klinikum der Johann-Wolfgang-Goethe-Universität<br>Medizinische Klinik II<br>Hämatologie und Onkologie<br>Theodor-Stern-Kai 7<br>60590 Frankfurt/Main	Prüfer:<br>Dr. Johannes Atta<br>Stellvertreterin:<br>Dr. Uta Brunnberg	Ethik-Kommission des Fachbereichs Medizin<br>der Johann Wolfgang Goethe-Universität Frankfurt<br>Theodor-Stern-Kai 7<br>60590 Frankfurt am Main
11	Universitätsklinikum Freiburg<br>Medizinische Klinik I<br>Hugstetter Str. 55<br>79106 Freiburg	Prüfer:<br>Prof. Dr. Jürgen Finke<br>Stellvertreterin:<br>Dr. Elisabeth Schorb	Ethik-Kommission<br>der Albert-Ludwigs-Universität Freiburg<br>Engelberger Straße 21<br>79106 Freiburg
12	Universitätsklinikum Göttingen<br>Abteilung Hämatologie/Onkologie, Neurochirurgie<br>Robert-Koch-Str. 40<br>37075 Göttingen	Prüfer:<br>Dr. med. Justin Hasenkamp<br>Stellvertreterin:<br>Dr. med. Friederike Braulke	Ethik-Kommission der Medizinischen Fakultät der<br>Georg-August-Universität<br>Von-Siebold-Straße 3<br>37075 Göttingen
13	Universitätsmedizin Greifswald<br>Klinik und Poliklinik für Innere Medizin C<br>Hämatologie, Onkologie und Transplantationszentrum<br>Sauerbruchstraße<br>17475 Greifswald	Prüfer:<br>Prof. Dr. Christian Andreas Schmidt<br>Stellvertreter:<br>Dr. Carsten Hirt	Ethikkommission an der<br>Universitätsmedizin Greifswald<br>Institut für Pharmakologie<br>Felix-Hausdorff-Str.3<br>17487 Greifswald
14	Universitätsklinikum Halle (Saale)<br>Klinik für Innere Medizin IV<br>Hämatologie/ Onkologie<br>Ernst-Grube-Straße 40<br>06120 Halle	Prüferin:<br>PD Dr. Katrin Jordan<br>Stellvertreter:<br>PD Dr. Lutz Müller	Ethik-Kommissionen der Medizinischen Fakultät<br>der Martin-Luther-Universität Halle-Wittenberg<br>Magdeburger Str. 16<br>06112 Halle (Saale)
15	Asklepios Klinik Altona<br>Hämatologie, internistische Onkologie und Palliativmedizin<br>Paul-Ehrlich-Straße 1<br>22763 Hamburg	Prüfer:<br>Prof. Dr. Christian Meyer zum Büschenfelde<br>Stellvertreter:<br>Dr. Murwan Ayoub	Ethik-Kommission der Ärztekammer Hamburg<br>Weidestraße 122 b<br>22083 Hamburg
16	Universitätskrankenhaus Hamburg-Eppendorf<br>Medizinische Klinik II<br>Onkologisches Zentrum<br>Martinistr. 52<br>20246 Hamburg	Prüferin:<br>PD Dr. Mascha Binder<br>Stellvertreter:<br>Dr. Winfried Alsdorf	Ethik-Kommission der Ärztekammer Hamburg<br>Weidestraße 122 b<br>22083 Hamburg
17	Medizinische Hochschule Hannover<br>Klinik für Hämatologie, Hämostaseologie, Onkologie und Stammzelltransplantation<br>Carl-Neuberg-Str. 1<br>30625 Hannover	Prüferin:<br>Prof. Dr. Anke Franzke<br>Stellvertreter:<br>PD Dr. Christian Könecke<br>Stellvertreterin:<br>PD Dr. Felicitas Thol	Ethik-Kommission der Medizinischen Hochschule Hannover<br>Carl-Neuberg-Straße 1<br>30625 Hannover
18	Universitätsklinikum des Saarlandes Homburg<br>Innere Medizin I<br>Kirrberger Straße<br>66424 Homburg/ Saar	Prüfer:<br>Prof. Dr. Michael Pfreundschuh<br>Stellvertreter:<br>Dr. Niels Murawski	Ethik-Kommission bei der Ärztekammer des Saarlandes<br>Faktoreistr. 4<br>66111 Saarbrücken

Participating centers und ethics committees

19	Medizinische Universitätsklinik Heidelberg<br>Abteilung Innere Medizin V<br>Im Neuenheimer Feld 410<br>69120 Heidelberg	Prüferin:<br>Prof. Dr. Gerlinde Egerer<br>Stellvertreter:<br>PD Dr. Mathias Witzens-Harig	Ethik-Kommission I<br>der Medizinischen Fakultät Heidelberg<br>Alte Glockengießerei 11/1<br>69115 Heidelberg
20	Universitätsklinikum Jena<br>Klinik und Poliklinik für Innere Medizin II<br>Hämatologie und internistische Onkologie<br>Erlanger Allee 101<br>07747 Jena	Prüfer:<br>PD Dr. Thomas Ernst<br>Stellvertreter:<br>PD Dr. Sebastian Scholl	Ethik-Kommission der Friedrich-Schiller-Universität Jena an der Medizinischen Fakultät<br>Bachstraße 18<br>07740 Jena
21	Universitätsklinikum Schleswig-Holstein, Campus Kiel<br>Klinik für Innere Medizin II<br>Hämatologie und Internistische Onkologie<br>Arnold-Heller-Str. 3 (Haus 50)<br>24105 Kiel	Prüfer:<br>Prof. Dr. Dr. Michael Kneba<br>Stellvertreterin:<br>PD Dr. Christiane Pott	Ethik-Kommission der Medizinischen Fakultät der Christian-Albrechts-Universität zu Kiel<br>Arnold-Heller-Straße 3<br>Haus 9<br>24105 Kiel
22	Universitätsklinikum Schleswig-Holstein<br>II. Medizinische Klinik und Poliklinik<br>Chemnitzstr. 33<br>24116 Kiel	Prüfer:<br>Prof. Dr. Dr. Michael Kneba<br>Stellvertreterin:<br>Dr. Monika Lamprecht	Ethik-Kommission der Medizinischen Fakultät der Christian-Albrechts-Universität zu Kiel<br>Arnold-Heller-Straße 3<br>Haus 9<br>24105 Kiel
23	Stiftungsklinikum Mittelrhein GmbH<br>Zentrum für Innere Medizin<br>Johannes-Müller-Str. 7<br>56068 Koblenz	Prüfer:<br>Prof. Dr. Ralph Naumann<br>Stellvertreter:<br>Dr. Dirk Niemann	Ethik-Kommission bei der Landesärztekammer Rheinland-Pfalz<br>Deutschhausplatz 3<br>55116 Mainz
24	Universitätsklinikum Köln<br>Innere Medizin 1<br>Kerpenerstr. 62<br>50937 Köln	Prüfer:<br>Prof. Dr. Peter Borchmann<br>Stellvertreter:<br>Prof. Dr. Kai Hübel<br>Stellvertreterin:<br>Dr. Stefanie Sasse	Ethik-Kommission der Medizinischen Fakultät der Universität zu Köln<br>Gebäude 5, Kerpener Str.62<br>50937 Köln
25	Universitätsklinikum Magdeburg AöR<br>Klinik für Hämatologie und Onkologie<br>Leipziger Str. 44<br>39120 Magdeburg	Prüfer:<br>Dr. Thomas Heinicke<br>Stellvertreter:<br>Dr. Enrico Schalk	Ethik-Kommission der Otto-von-Guericke-Universität<br>an der Medizinischen Fakultät<br>Leipziger Straße 44<br>39120 Magdeburg
26	Universitätsmedizin der Johannes Gutenberg-Universität<br>III. Med. Klinik und Poliklinik<br>Langenbeckstr. 1<br>55101 Mainz	Prüfer:<br>PD Dr. Georg Heß<br>Stellvertreter:<br>Dr. Markus Munder	Ethik-Kommission bei der Landesärztekammer Rheinland-Pfalz<br>Deutschhausplatz 3<br>55116 Mainz
27	Klinikum der Universität München - Großhadern<br>Medizinische Klinik und Poliklinik III<br>Hämatologie/Onkologie<br>Marchioninistr. 15<br>81377 München	Prüfer:<br>Prof. Dr. Martin Dreyling<br>Stellvertreterin:<br>Dr. Louisa von Baumgarten	Ethik-Kommission der Medizinischen Fakultät<br>der Ludwig-Maximilians Universität, München<br>Pettenkoferstr. 8a<br>80336 München

Participating centers und ethics committees

28	Klinikum rechts der Isar<br>der TU München<br>III. Med. Klinik und Poliklinik<br>Hämatologie u. Internistische Onkologie<br>Ismaninger Straße 22<br>81675 München	Prüfer:<br>Prof. Dr. Ulrich Keller<br>Stellvertreter:<br>Dr. Alexander Esmaty	Ethik-Kommission der Fakultät<br>für Medizin der Technischen Universität<br>München<br>Ismaninger Str. 22<br>81675 München
29	Ethik-Kommission der<br>Ärztekammer Westfalen-Lippe<br>und der Westfälischen Wilhelms-<br>Universität Münster<br>Gartenstraße 210-214<br>48147 Münster	Prüfer:<br>Prof. Dr. Georg Lenz<br>Stellvertreterin:<br>Dr. Andrea Kerkhoff	Ethik-Kommission der Ärztekammer<br>Westfalen-Lippe und der Westfälischen<br>Wilhelms-Universität Münster<br>Gartenstraße 210-214<br>48147 Münster
30	Pius-Hospital<br>Abteilung für Internistische<br>Onkologie<br>Georgstraße 12<br>26121 Oldenburg	Prüfer:<br>Prof. Dr. Frank Griesinger<br>Stellvertreterin:<br>Dr. Imme Conradi	Ethik-Kommission bei der<br>Ärztekammer Niedersachsen<br>Berliner Allee 20<br>30175 Hannover
31	Klinikum Oldenburg gGmbH<br>Abt. Onkologie/Hämatologie<br>Rahel-Straus-Str. 10<br>26133 Oldenburg	Prüfer:<br>Prof. Dr. Bernd Metzner<br>Stellvertreterin:<br>Dr. Ruth Thole	Ethik-Kommission bei der<br>Ärztekammer Niedersachsen<br>Berliner Allee 20<br>30175 Hannover
32	Universitätsklinikum Regensburg<br>Klinik & Poliklinik für Innere<br>Medizin III<br>Hämatologie & Onkologie<br>Franz-Josef-Strauss-Allee 11<br>**93053 Regensburg**	Prüfer:<br>Prof. Dr. Tobias Pukrop<br>Stellvertreter:<br>Dr. Joachim Hahn	Ethik-Kommission an der Universität<br>Regensburg<br>Franz-Josef-Strauß Allee 11<br>93053 Regensburg
33	Universitätsklinikum Tübingen<br>Medizinische Klinik II<br>Otfried-Müller-Str. 10<br>72076 Tübingen	Prüfer:<br>Prof. Dr. Robert Möhle<br>Stellvertreter:<br>Dr. Martin Sökler	Ethik-Kommission der Medizinischen<br>Fakultät und am Universitätsklinikum<br>Tübingen<br>Gartenstraße 47<br>72074 Tübingen
34	Universitätsklinikum Ulm<br>Klinik für Innere Medizin III<br>Hämatologie, Onkologie,<br>Rheumatologie und<br>Infektionskrankheiten<br>Albert-Einstein-Allee 23<br>89081 Ulm	Prüfer:<br>Prof. Dr. Stephan Stilgenbauer<br>Stellvertreterin:<br>Dr. Henriette Huber	Ethik-Kommission der Universität Ulm<br>Helmholtzstraße 20<br>89081 Ulm
35	Schwarzwald-Baar Klinikum<br>Villingen-Schwenningen GmbH<br>Klinikstr. 11<br>78052 Villingen-Schwenningen	Prüfer:<br>Prof. Dr. Wolfgang Brugger<br>Stellvertreterin:<br>Dr. Anja Rückert	Ethik-Kommission bei der<br>Landesärztekammer<br>Baden - Württemberg<br>Jahnstraße 40<br>70597 Stuttgart
