# Supplementary material for: High-dose chemotherapy and autologous stem cell transplant compared with conventional chemotherapy for consolidation in newly diagnosed primary CNS lymphoma—a randomized phase III trial (MATRix)
Source: BMC Cancer. 2016 Apr 21;16:282. doi: 10.1186/s12885-016-2311-4 (PMC4839072; doi:10.1186/s12885-016-2311-4)
Supplement: Additional file 2: — SPIRIT checklist. (PDF 552 kb) [file 12885_2016_2311_MOESM2_ESM.pdf]

SPIRIT
STANDARD PROTOCOL ITEMS: RECOMMENDATIONS FOR INTERVENTIONAL TRIALS

SPIRIT 2013 Checklist: Recommended items to address in a clinical trial protocol and related documents*

Section/item	Item No	Description
**Administrative information**		
Title	1	High-dose chemotherapy and autologous stem cell transplant or consolidating conventional chemotherapy in primary CNS lymphoma - randomized phase III trial - MATRix / IELSG43
Trial registration	2a	German clinical trials registry DRKS00005503 (registered 22 April 2014)<br>ClinicalTrials.gov NCT02531841 (registered 24 August 2015)
	2b	NCT-No.: NCT02531841
Protocol version	3	CTP_V02_09.12.2015
Funding	4	Federal Department of Education and Research (BMBF/DLR), Riemser Pharma GmbH
Roles and responsibilities	5a	Study chairman Germany:<br>Prof. Dr. Gerald Illerhaus<br>Clinic of Hematology, Oncology and Palliative Care, Klinikum Stuttgart, Stuttgart, Germany<br><br>Study chairman for all non-German sites on behalf of the IELSG43:<br>Andrés J. M. Ferreri<br>Unit of Lymphoid Malignancies, Head Division of OncoHematological Medicine, Department of OncoHematology, San Raffaele Scientific Institute, Milan, Italy<br><br>Biostatstician<br>Dr. rer. nat. Gabriele Ihorst<br>Clinical Trials Unit, Freiburg University Medical Center, Freiburg, Germany<br><br>Medical Trial Coordinator<br>Dr. med. Elisabeth Schorb<br>Department of Hematology / Oncology, Freiburg University Medical Center, Freiburg, Germany

Randomization/Registration Office
Clinical Trials Unit, Freiburg University Medical Center, Freiburg, Germany

Pharmacovigilance/SAE Managment for all German sites
Clinical Trials Unit, Freiburg University Medical Center, Freiburg, Germany

Project Coordinator
Elvira Burger
Clinical Trials Unit, Freiburg University Medical Center, Freiburg, Germany

Monitoring for all German Sites
Heidi Fricker
Department of Hematology / Oncology, Freiburg University Medical Center, Freiburg, Germany

Data managment
Clinical Trials Unit, Freiburg University Medical Center, Freiburg, Germany

Reference center radiology for all German sites
Dr. Claudia Harder
Medical Center Kantonspital St. Gallen
Institute for Radiology, St. Gallen, Switzerland

Reference center radiology for all IELSG sites
Andres Falini
Department of Neuroradiology, San Raffaele H Scientific Institute, Milan, Italy

Reference center pathology for all German sites
Prof. Dr. Martina Deckert, University Medical Center Koeln, Institute for Neuropathology, Koeln, Germany

Reference center pathology for all IELSG sites
Maurilio Ponzoni, Unit of Lymphoid Malignancies Pathology Unit, San Raffaele H Scientific Institute, Milan, Italy

Data Monitoring Committee (DMC):
1. Prof. Dr. Hendrik-Johannes Pels (Neurologist)
Medical Center Barmherzige Brüder Regensburg, Germany2. Prof. Dr. Lorenz Truemper (Hematologist)
Center for Internal Medicine, Medical Department for Hematology and Oncology, Goettingen, Germany
3. Dr. rer.nat. Geraldine Rauch (Biostatistician)

		Medical University Hospital Heidelberg, Medical Biometry and Informatics, Heidelberg, Germany
	5b	Stuttgart, represented by the<br>Executive Medical Director<br>Prof. Dr. J. Graf<br>Medical Center Stuttgart
	5c	Study Sponsor will be responsible for study design, collection management, interpretation of data, writing of the report and the decision to submit the report for publication. Sponsor has ultimate authority over any of these activities.
	5d	Composition, roles, and responsibilities of the coordinating centre, endpoint adjudication committee, data management team, and other individuals or groups overseeing the trial, not applicable see Item 21a for data safety monitoring committee.
**Introduction**		
Background and rationale	6a	Scientific background: Primary CNS lymphoma (PCNSL) accounts for 1 to 2% of all Non-Hodgkin's lymphomas (NHL) and for 2 to 7% of all primary CNS tumors. Its incidence has increased over the past 30 years, particularly in immunocompetent individuals. Over 90% of PCNSL are lymphomas of B-cell origin, accounting to the subtype diffuse large B-cell lymphoma (DLBCL). Prognosis without treatment resembles that of systemic high-grade NHL, and the median survival of untreated patients with PCNSL is approximately 3 months. Even though therapy has improved the outcome of PCNSL patients remains unsatisfactory when compared to patients with extra-CNS-NHL. Many questions about what constitutes the optimal therapeutic approach remain unanswered, and these must be addressed in future trials.<br>Historically, radiotherapy (RT) has been the standard treatment for PCNSL with response rates of 60 to 97%, a median survival of 14 months, and a 5-year survival of 3 to 26% (1). Despite the high CR rates, almost all patients treated only with RT relapse after a few months (2). Initial improvements were achieved with the combination of HD-MTX-based chemotherapy and WBRT leading to median survival times of 36 to 60 months (3-5). Therefore, the addition of chemotherapy to RT has been recommended to improve the survival of PCNSL patients (6, 7). The superiority of a combined strategy is also reinforced by three large retrospective multicenter surveys reporting therapeutic results in over 1000 patients treated in Europe and Japan (6, 8). These studies uniformly showed that HD-MTX is the most efficient known cytostatic agent, while any regimen including all other standard regimens for treating extra-CNS malignant lymphoma

without HD-MTX are associated with outcomes no better than with RT alone. Several attempts were made to optimize chemotherapy in PCNSL. As the most active drugs and regimens such as anthracycline- and cyclophosphamide-based therapies yielded unsatisfactory results due to their incapacity to cross the blood brain barrier (BBB) several attempts were made to combine the most active agent (HD-MTX) with other drugs penetrating the BBB. A first randomized phase II trial with completed recruitment on primary chemotherapy in PCNSL documented the clear superiority of combining high-dose cytarabine (HD-Ara-C) and HD-MTX compared to HD-MTX alone.(9) Complete and partial remission rates in the MTX/Ara-C arm were 46% and 23%, compared to 18% and 23% in the HD-MTX arm, respectively. Failure-free survival and OS were higher in the combined treatment group. At a median follow-up of 30 months (range 12 to 55), the HD-MTX group's 3-year FFS was 21% and 38% in the MTX/Ara-C group (p=0.01), with 3-year overall survival of 32% and 46%, respectively, (p=0.07). In a follow-up analysis OS was significantly higher with extended 46-month follow-up (45% vs. 24%; p=0.05) [personal communication with A. Ferreri, IELSG-Group]. Other agents such as lomustine, procarbacine, vincalkaloids, temozolomide and thiotepa have also been added to HD-MTX showing promising remission rates and acceptable toxicity profiles; however randomized trials to prove the superiority of these combinations over HD-MTX alone are lacking and should be initiated in the future. Rituximab is a standard agent for treating systemic B-cell lymphomas.(10) However, in PCNSL, although already in wide use, the value of rituximab rests mainly on evidence from systemic lymphoma trials, but is now under investigation in two large ongoing randomized PCNSL studies (NCT01011920, NTR2427).

The role of high-dose chemotherapy followed by autologous stem-cell transplant (HDT-ASCT) has been investigated in several phase II trials for primary, relapsed, or refractory PCNSL and revealing promising results concerning response and survival rates.(11-15) However, randomized trials demonstrating a benefit of this concept over conventional optimized combination chemotherapy have not yet been carried out and are therefore needed. Thus the MATRix trial described herein is designed to determine whether HDT-ASCT is superior to conventional therapy as consolidation after intensified immunochemotherapy in newly diagnosed PCNSL.

The efficacy of WBRT (the current standard for consolidation after HD-MTX-based systemic treatment) is being compared to HDT-ASCT in the ongoing IELSG-32 trial. A similar question has been addressed in the current "Freiburger ZNS-NHL-Studie" focused on efficacy of the upfront transplantation, which recently finished recruitment.

Evidence - clinical trials: High-dose chemotherapy and ASCT are known to be a highly efficient treatment strategy for NHL. In the pre-

rituximab-era it was the therapeutic backbone for the treatment of relapsed and refractory DLBCL as well as first-line treatment for high-risk DLBCL. Treatment of PCNSL differs from that in other DLBCL-locations due to the fact that most of the active drugs in NHL-treatment cannot pass the BBB. This problem can be overcome by using drugs penetrating the CNS and/or by increasing the doses in order to reach higher drug-levels in the CNS. The most active chemotherapeutic drug in the treatment of PCNSL is HD-MTX. Beside protocols applying HD-MTX as a single agent, various treatment regimens containing additional chemotherapeutics have been proposed, but the only agent yet tested in a randomized trial is cytarabine, which - in addition to HD-MTX - leads to improved response and survival rates.(9) Other combinations have been investigated, of which thiotepa-containing schedules have revealed promising response and survival rates. A single-arm phase II trial assessing the chemotherapy combination named “MATILDE” included thiotepa.(16) This combination was associated with an overall response rate of 72% and a CR rate of 46% with a 5-yr OS of 42% and a persisting plateau in the survival curve. The use of thiotepa seems justified by its great bioavailability in the CNS, its high efficacy in aggressive lymphoma and in recently reported trials on PCNSL.(11, 12, 16) Based on experience with other hematological malignancies and the need for effective consolidation treatment, HDT-ASCT was also evaluated in PCNSL. The rationale for the impact of HDT-ASCT in PCNSL is its delivery into the CNS of blood-brain-barrier (BBB) penetrating agents at several-fold higher concentrations than conventional therapy, which cannot provide such penetration. (17, 18) In recent trials we demonstrated a high rate of continuous remissions after treatment with HDT-ASCT with or without WBRT. In a first pilot and phase-II study, we treated 30 patients with PCNSL ≤ 65 years with sequential induction chemotherapy including three cycles of HD-MTX, HD-AraC, and thiotepa followed by stem-cell harvest. The conditioning regimen consisted of carmustine and thiotepa followed by ASCT; WBRT was given as consolidation.(11) Twenty-three of the 30 patients proceeded to HDT-ASCT resulting in CR and PR in 15 and 8 patients, respectively. Twenty-one patients subsequently underwent WBRT and all achieved CR. With a median follow-up of 63 months, the 5-year OS was 69% for all patients and 87% for those completing HDT and ASCT, respectively. Five-year relapse-related mortality was 21% for all patients (n=30) and 8.7% for those 23 treated with HDT and ASCT, respectively. In a follow-up pilot study, induction chemotherapy was intensified, the thiotepa dose was doubled, and only those patients not achieving CR after induction therapy underwent WBRT.(12) Seven of eleven patients were in CR following ASCT, and 3 in PR upon ASCT received radiotherapy as consolidative treatment. After a median follow-up of 25 months, 3-year OS was 77%. None of the patients suffered from severe neurotoxicity

during the follow-up period. Both trials suggest a curative effect of HDT-ASCT in young PCNSL patients. This concept, supplemented by rituximab immunotherapy, was evaluated in a phase II trial ("Freiburg ZNS-NHL Trial", ClinicalTrials.gov Identifier: NCT00647049).

Preliminary results showed an overall remission rate (ORR) for the intention-to-treat population of 91% (77% CR and 14% PR), for patients treated with HDT and ASCT (n=73) ORR was 91%.(19) After a median follow-up of 35 months, 3-year overall survival was 77.6% for all patients and 87.1% for patients after HDT.

In light of these positive findings, we initiated an ongoing international randomized phase-II trial (2 randomizations, 1st 3 arms, 2nd 2 arms) in collaboration with the International Extranodal Lymphoma Study Group (IELSG) on primary chemotherapy with HD-MTX and HD-Ara-C with or without thiotepa, and with or without rituximab (1st randomization), followed by whole brain radiotherapy vs. high-dose chemotherapy supported by autologous stem-cell transplantation (2nd randomization) for immunocompetent patients with newly-diagnosed primary CNS lymphoma (ClinicalTrials.gov Identifier: NCT01011920). In this trial, we anticipate being able to determine what constitutes the best induction treatment, as well as the superiority of HDT-ASCT or WBRT as consolidation treatment. Pilot patients have shown, apart from expected hematotoxicity, good tolerability of the combination of rituximab, MTX, Ara-C and thiotepa. This multicenter trial is being conducted in Italy, Germany, Switzerland and the United Kingdom, and is being co-chaired equally by Andres Ferreri and Gerald Illerhaus. Gerald Illerhaus is the Principal Investigator of the German centers.

The "Freiburg Protocol" for HDT-ASCT as well as the "IELSG-32 Trial" are both being conducted by the "Cooperative Primary CNS-Lymphoma Study Group Freiburg" with more than 20 medical centers in Germany.

Several trials have demonstrated that consolidating strategies with non-cross-resistant cytostatic agents in first-line treatment yielded promising results in the treatment of PCNSL. In a recently published trial, etoposide in combination with cytarabine as consolidation after an induction therapy with HD-MTX, rituximab and temozolamide showed encouraging results with a 2-year time to progression of 77% in patients who completed consolidation.(20) Another regimen with dexamethasone, etoposide, ifosfamide and carboplatin (DeVIC) revealed high response rates in patients with PCNSL.(21)

6b Explanation for choice of comparators

The current standard recommendation for previously untreated PCNSL is the combination of HD-MTX and HD-Ara-C followed by WBRT.(9) In order to improve the response, the combination of HD-MTX/HD-Ara-C with thiotepa and rituximab was investigated in

consecutive trials demonstrating feasibility and high efficacy (19) ClinicalTrials.gov Identifier: NCT01011920. Therefore we chose the combination of rituximab, HD-MTX, HD-Ara-C and thiotepa (4 cycles), which is assumed to be the most effective arm of the IELSG-32-trial as induction treatment.

In view of the high efficacy of the Freiburg Protocol high-dose chemotherapy with BCNU and thiotepa and autologous stem-cell transplantation was chosen as high-dose consolidation (arm B).

For the conventional consolidation (arm A) we chose a treatment of 2 cycles of R-DeVIC. The DeVIC combination chemotherapy regimen has been applied in recurrent and refractory as well as in newly diagnosed PCNSL. A retrospective analysis of 21 patients with newly-diagnosed PCNSL who received DeVIC chemotherapy followed by WBRT showed high efficacy and a good safety profile.(21) Overall response rate was 95.2% in newly diagnosed PCNSL and 83% in refractory and recurrent PCNSL. Median progression-free survival (PFS) time in newly-diagnosed PCNSL was 37.4 months, median OS time 47.8 months. The most frequent grade 3-4 adverse events related to DeVIC chemotherapy are bone marrow suppression, appetite loss, stomatitis, gastrointestinal side effects, hypokalemia and hyponetremia and about 9% grade 4 febrile neutropenias. The most common non-hematologic toxicity related to DeVIC chemotherapy was pretibial edema. All side effects were manageable, and no treatment-related deaths have been reported so far. In patients receiving WBRT after DeVIC chemotherapy important neurotoxic effects were observed in 31.6%.(21, 22)

Objectives	7	Comparison of high-dose chemotherapy followed by autologous stem cell transplantation with optimized conventional chemotherapy regarding PFS, OS, treatment response (rate of complete responses) and treatment related morbidities (neurotoxicity and adverse advents) in patients with primary CNS lymphoma.
Trial design	8	This is a randomized, controlled, open-label, multicenter phase III trial with two parallel arms.

Methods: Participants, interventions, and outcomes

Study setting	9	We recruit patients from approximately 35 hospitals in Germany. Furthermore, the International Extranodal Lymphoma Study Group (IELSG) will participate in the study and recruit patients. If necessary, additional qualified sites can be included during the course of the trial. For a list of the currently recruiting centers in Germany please refer to the Appendix section.

Eligibility criteria 10

Inclusion criteria:

1. Immunocompetent patients with newly-diagnosed primary central nervous system B-cell lymphoma
2. Age 18-65 years irrespective of ECOG or 66-70 years
3. (with ECOG Performance Status ≤ 2)
4. Histologically or cytologically assessed diagnosis of B-cell lymphoma by local pathologist. Diagnostic sample obtained by stereotactic or surgical biopsy, CSF cytology examination or vitrectomy
5. Diagnostic sample obtained by stereotactic or surgical biopsy, CSF cytology examination or vitrectomy
6. Disease exclusively located in the CNS
7. At least one measurable lesion
8. Previously untreated patients (previous or ongoing steroid treatment admitted)
9. Sexually active patients of childbearing potential who agree to take adequate contraceptive measures during study participation
10. Written informed consent obtained according to international guidelines and local laws by patient or authorized legal representative in case patient is temporarily legally not competent due to his or her disease

Additional randomization criteria:

11. Sufficient stem cell harvest (≥ 5 x 106 CD34+ cells/kg of body weight)
12. Complete remission, unconfirmed complete remission or partial remission
13. Central pathology results confirming local results.

Exclusion criteria:

1. Congenital or acquired immunodeficiency
2. Systemic lymphoma manifestation (outside the CNS)
3. Isolated ocular lymphoma without manifestation in the brain parenchyma or spinal cord
4. Previous or concurrent malignancies with the exception of surgically cured carcinoma in-situ of the cervix, carcinoma of the skin or other kinds of cancer without evidence of disease for at least 5 years
5. Previous Non-Hodgkin lymphoma at any time
6. Inadequate bone marrow (platelet count decreased ≥CTC grade 1, anemia ≥CTC grade 1, neutrophil count decreased ≥CTC grade 1), renal (creatinine clearance < 60 ml/min), cardiac

(ejection fraction decreased ≥CTC grade 2), or hepatic function (blood bilirubin increased ≥CTC grade 2, alanine aminotransferase increased ≥CTC grade 2, aspartate aminotransferase increased ≥CTC grade 2 or gamma-GT increased ≥CTC grade 2)

7. HBsAg, anti-HBc or HCV positivity
8. HIV infection, previous organ transplantation or other clinical evident form of immunodeficiency
9. Concurrent treatment with other experimental drugs or participation in a clinical trial within the last thirty days before study inclusion
10. Symptomatic coronary artery disease, cardiac arrhythmias uncontrolled with medication or myocardial infarction within the last 6 months (New York Heart Association Class III or IV heart disease)
11. Severe non-compensated pulmonary disease (IVC <55%, DLCO <40%)
12. Third space fluid accumulation > 500 ml
13. Hypersensitivity to study treatment or any component of the formulation
14. Taking any medications likely to cause interactions with the study medication
15. Known or persistent abuse of medication, drugs or alcohol
16. Patient without legal capacity and who is unable to understand the nature, significance and consequences of the study and without designated legal representative
17. Persons who are in a relationship of dependency/employment to the sponsor and/ or investigator
18. Any familial, sociological or geographical condition potentially hampering compliance with the study protocol and follow-up schedule
19. Concurrent (or planned) pregnancy or lactation
20. Fertile patients refusing to use safe contraceptive methods during the study

Interventions	11a	All enrolled patients will receive induction chemotherapy with rituximab, HD-MTX, HD-Ara-C and thiotepa (MATRix regimen). The maximum number of induction chemotherapy courses will be four. Chemotherapy will be administered every three weeks. Patients in complete remission (CR), unconfirmed complete remission (CRu), partial remission (PR) or stable disease (SD) after 2 courses will receive additional two courses of the primary chemotherapy regimen. Stem-cell harvest will be performed after the second course for all

patients. Response assessment by brain MRI will be done after the second and the fourth course. Patients not achieving CR, CRu, PR or SD after the second course, and patients not achieving CR, CRu or PR after the fourth course, as well as those who experience progressive disease (PD) at any time will be assigned to off-study salvage therapy. Patients whose stem-cell harvest is insufficient after three cycles of induction treatment will be treated off-study as well. After 4 cycles of induction chemotherapy, the inclusion and exclusion criteria will be re-evaluated. Only those patients achieving PR or CR after 4 cycles of induction chemotherapy are eligible for randomization. The enrolled patients will be randomized to undergo conventional consolidation with R-DeVIC (arm A) or consolidating high-dose chemotherapy with BCNU (or busulfan if BCNU is not available) and thiotepa followed by ASCT (arm B).

11b Discontinuation criteria for the individual patient: In case of PD at any time before randomization, SD after four cycles of induction chemotherapy, insufficient stem-cell harvest, inadequate bone marrow recovery (discontinuation of chemotherapy in case of delay of more than four weeks) before randomization or toxicity before randomization, trial treatment is terminated prematurely.

Investigators should follow the guidelines below for dose modification of treatment with investigational product; any deviation must be discussed previously with the sponsor unless it concerns a patient's safety. All dose changes or interruptions must be recorded on the appropriate CRF page.

In case of inadequate bone marrow recovery, that is ANC <1.500/µl (<1.200/µl in arm "A") and platelets <90.000/µl, on the intended day of re-treatment, the start of the next cycle can be delayed for a maximum of 4 weeks. Thereafter, chemotherapy must be discontinued, and patients will be treated according to the investigator's decision.

In case of non-hematologic toxicity dose modification should be managed according to the investigator`s judgement and as recommended in the drug information. Rituximab infusion reactions will be managed according to international guidelines.

11c Strategies to improve adherence to intervention protocols, and any procedures for monitoring adherence (eg, drug tablet return, laboratory tests)

Information about drugs given to the patient must be entered in the source documents and in the CRFs to accurately determine the patient's drug exposure throughout the trial.

Drug accountability will be performed according to the routine pharmacy practice without study specific documentation in the Investigator Site/Pharmacy File.

11d Permitted medication: Patients with newly-diagnosed PCNSL often receive corticosteroids to reduce perifocal brain edema. However, whenever possible, steroids should be tapered out as early as possible before biopsy or at the very latest, once chemotherapy has started. If patients suffer from epilepsy due to lymphoma manifestations, anti-epileptic drugs are allowed and can be continued as clinically indicated. The patient must notify the investigational site about any new medications he or she is taking after starting the trial medication. The following drugs can be applied in our trial: antiemetics, analgesics, antibiotics, anticonvulsants, sedatives, antihyperuricemic agents as well as other therapies to control metabolic and malnutrition disturbances. Corticosteroids during treatment and their definitive interruption will depend on clinical requirements. If possible, corticosteroids should be tapered out once chemotherapy has started. It is important that the type and doses of corticosteroids be accurately recorded. Oral antiviral, antifungal and antipneumocystic prophylaxis is strongly suggested. Antimicrobial drugs should be interrupted during chemotherapy administration to avoid potentially negative pharmacological interactions. Rituximab infusion reactions will be managed according to international guidelines. Folinic-acid rescue starts 24 hours after the start of MTX infusion and is recommended to be scheduled and documented according to the attached leucovorin rescue sheet. The post-MTX hydration should attain a total volume of 2000 ml. All further medications during the trial must be documented in the patient file, as well as the indication, dosage and period of administration. All medications not triggering known interactions can be given during the trial if necessary and clinically indicated. The following drugs can be applied in our trial: antiemetics, analgesics, antibiotics, anticonvulsants, sedatives, antihyperuricemic agents as well as other therapies to control metabolic and malnutrition disturbances. Corticosteroids during treatment and their definitive interruption will depend on clinical requirements. If possible, corticosteroids should be tapered out once chemotherapy has started. It is important that the type and doses of corticosteroids be accurately recorded. Oral antiviral, antifungal and antipneumocystic prophylaxis is strongly suggested. Antimicrobial drugs should be interrupted during chemotherapy administration to avoid potentially negative pharmacological interactions. Rituximab infusion reactions will be managed according to international guidelines. The post-MTX hydration should attain a total volume of 2000 ml. All further medications during the trial must be documented in the patient file, as well as the indication, dosage and period of administration. All medications not triggering known interactions can be given during the trial if necessary and clinically indicated.

Additional cytotoxic therapy, biological responsive modifiers and drugs possibly interfering in the action or pharmacokinetics of rituximab,

		MTX, Ara-C, thiotepa, dexamethasone, etoposide, ifosfamide, carboplatin or BCNU (busulfan where necessary) must be avoided.
Outcomes	12	The primary objective of this study is to demonstrate the efficacy measured as progression-free survival (PFS) of intensive chemotherapy followed by autologous stem-cell transplantation compared to conventional chemo-therapy. PFS is defined as the time from randomization until PD or relapse or death from any cause.<br>Secondary objectives are<br>1. To compare HDT-ASCT with optimized conventional chemotherapy regarding CR. CR will be determined on day 60 after randomization.<br>2. To compare HDT-ASCT with optimized conventional chemotherapy regarding response duration. Response duration is defined as time from CR, CRu or PR until relapse or PD.<br>3. To compare HDT-ASCT with optimized conventional chemotherapy regarding OS over time. OS is defined as time from randomization until death of any cause.<br>4. To compare HDT-ASCT with optimized conventional chemotherapy regarding quality of life (QOL) over time. Quality of life is measured with the EORTC QLQ-C30.<br>5. To compare HDT-ASCT with optimized conventional chemotherapy regarding safety. The safety endpoint are (Serious) adverse events<br>6. To compare HDT-ASCT with optimized conventional chemotherapy regarding toxicity. Toxicity is recorded in the toxicity tables<br>7. To compare HDT-ASCT with optimized conventional chemotherapy regarding neurotoxicity. Neurotoxicity is measured with the minim mental status examination, the EORTC QLQ-BN20 questionnaire and the neuro-psychological battery.
Participant timeline	13	Please refer to the Appendix Section (Study Flow Chart)
Sample size	14	Sample size calculation is based on the primary endpoint PFS. We are assuming that the PFS rate for patients treated with the conventional intervention (arm A) is approximately 50% after 3 years. To compare the two treatment groups, a hazard ratio of 1.8 of the conventional intervention vs. the high-dose treatment (arm B) is considered to be clinically relevant. This corresponds to a PFS rate after 3 years of 68% in the high-dose group, which is considered as realistic. In order to detect a difference between arm A and arm B with a power of 80% at a two-sided significance level of 5% under this assumption, a total number of 92 events is required. Assuming an exponential model for survival, an accrual period of 3 years and an additional follow-up time of 2 years, 200 patients should be included.<br>As follow-up may be incomplete for a few patients, 220 patients will be randomized. Furthermore, we anticipate that some patients (about 10%) will fail to attain complete or partial remission during the first 4

		chemotherapy cycles (start induction treatment) and will therefore not be randomized. We therefore assume that approximately 250 patients will need to be included in the study (start induction treatment).
Recruitment	15	35 leading German hematological and neuro-oncological centers are already participating in the trial. In order to increase recruitment rate the IELSG is going to participate with more than 40 centers soon.

Methods: Assignment of interventions (for controlled trials)

Allocation: Sequence generation	16a	A randomized design (block randomization with randomly-varying block sizes with an allocation ratio of 1:1) is applied in order to ensure comparability of the treatment groups. Central randomization by fax will be performed to guarantee concealment of the treatment allocation. Stratification according to response status (CR or PR) after 4 courses of induction chemotherapy will be performed. Patients with SD or PD after induction treatment will be treated off-study. No stratification by study centers will take place, because many centers having small numbers of patients will be included in the trial. Study site-initiated stratification could create problems with the statistical analysis (unstable treatment effect estimation). See EMA Points to Consider on Adjustment for Baseline Covariates, section II.3, CPMP/EWP/2863/99. Randomization will take place after 4 cycles of induction therapy, i.e. immediately before starting treatment with either HDT-ASCT or R-DeVIC in order to enable an analysis according to the intention-to-treat (ITT) principle with as few protocol violators or drop-outs as possible.<br>The block lengths will be documented separately and will not be disclosed to the centers. The randomization code will be produced by validated programs based on the Statistical Analysis System (SAS®).
Allocation: Concealment mechanism	16b	The patient identification code - the same number as in the registration - will be entered on the randomization form and the questions on re-checking the inclusion/exclusion criteria on the form will be answered. The fully completed form will then be faxed to the CTU for randomization (the same contact data as for registration see above). The CTU will review the patient's details on the randomization fax and randomize the patient. If the details on the randomization fax appear incomplete or implausible, the CTU will send the investigator a query fax for clarification.
Implementation	16c	Patients will be enrolled by the local investigators. The treatment will be initiated by the local investigator according to the randomized treatment arm.

Blinding (masking)	17	not applicable

Methods: Data collection, management, and analysis

Data collection methods	18a	The investigator or anyone designated by the investigator shall document the trial data on a trial-specific case report form (CRF) as promptly as possible. Hard-copy CRFs will be used in this trial. The hard copy CRFs consist of 3-layer Non-Carbon-Required paper (NCR paper). All data collected during the trial will be entered on the trial-specific CRF pages by the responsible investigator, or a person designated by the investigator.<br>Hematological tests and blood chemistry are recommended to be done twice a week during the therapy. Laboratory data must be checked by the investigator.<br>The following laboratory data are to be documented as toxicity parameters and graded according to CTCAE 4.0: Hematology includes assessment of white blood count (WBC), neutrophils, hemoglobin, and platelets. Blood chemistry comprises creatinine, total bilirubin, ALT, AST, and gamma-GT (only at screening).<br>During therapy, creatinine must be documented in the CRF in the measured unit to permit approximation of the kidney glomerular filtration rate (GFR) using the MDRD (Modification of Diet in Renal Disease) formula. Lactate dehydrogenase will be documented at screening by indicating "not increased" or "increased".<br>Quality of Life Questionnaire is to be filled out by the patient him/herself. It is important that the investigator not influence the patient in any way. The European Organization for Research and Treatment of Cancer QOL Questionnaire-30 (EORTC QLQ-C30) and Brain Cancer Module-20 (EORTC QLQ-BN20) are used. The EORTC QLQ-C30 measures five functional scales and global QOL. The EORTC QLQ-BN20 assesses items such as visual disorders, communication deficit, and future uncertainty. A standardized test battery designed to evaluate neurotoxicity in multi-national PCNSL trials (23) is to be used if available at the investigation site. Neuropsychological evaluation is done in about 30-60 minutes. The tests measure attention and executive function as well as motor skills and are performed at screening, the EOT visit, and annually thereafter with documentation in the CRF.
	18b	Screening failures are defined as patients who signed an ICF but failed to be registered in the study for any reason. These patients are to be documented on the subject screening log. For these patients, the screening CRFs and CRF pages with inclusion/exclusion criteria must be completed and "screening failure" stated on the registration fax naming at least one inclusion criteria not fulfilled or one exclusion criteria present. In case a patient's trial treatment has been stopped prematurely before randomization, all CRF pages until the time of termination must be completed including the premature end of

treatment" page and "re-check of inclusion and exclusion criteria". To document a patient's survival and remission status, the follow-up pages for non-randomized patients indicating patient "dead" or "alive" and "remission status" must also be completed.
In case a patient's trial treatment has been stopped prematurely after randomization, all CRF pages must be completed including the "premature end of treatment" page and regular follow-up pages.

Data management	19	The investigator will record participation in the trial, the frequency of trial visits, relevant medical data, concomitant treatment/s and the occurrence of adverse events in each trial patient's medical record. The investigator or anyone designated by the investigator shall document the trial data on a trial-specific case report form (CRF) as promptly as possible. Hard-copy CRFs will be used in this trial. The hard copy CRFs consist of 3-layer Non-Carbon-Required paper (NCR paper). For further details please refer to the CRF completion Instructions. All data collected during the trial will be entered on the trial-specific CRF pages by the responsible investigator, or a person designated by the investigator. Corrections and subsequent changes to CRF pages must be made according to the ICH-GCP guidelines provided in the CRF Completion instructions at the beginning of each CRF-folder. The study data will be managed using the DAMAST Version 9.2, a proprietary data management system based on the software package SAS®, which has been developed, validated and is maintained by the Clinical Trials Unit (CTU). Details on data management (procedures, responsibilities, data corrections, which may be made by Data Management staff themselves) will be described in a data management manual (DMM) prior to the trial. The data management manual is a working document and also contains a record of all data management processes carried out during the clinical trial and deviations of version 01 of the DMM. Before any data is entered, the trial database will be validated and specifications of the database documented in a variables handling plan. Double data entry will be performed by two different persons (with the exception of free text). The comparison of both entries and resolution of discrepancies may only be done by trained staff. An audit trail will be created to provide an electronic record of which data were entered or subsequently changed by whom and when. SAS® software will be used to review the data for completeness, consistency and plausibility. The checks to be programmed will be specified beforehand in a data validation plan. After running the check programs, the resulting queries will be sent to the investigator for review of his/her data. Answered queries will also be entered twice, verified and the updated data will then be transferred to the database. All programs which can be used to influence the data or the data quality will be validated (e.g. check programs, programs used for the input of external data, etc.).

		Data coding: Concomitant treatments or procedures entered into the database will be coded using the WHO Drug Reference List, or the Anatomical Therapeutic Chemical classification system provided by the WHO. All AEs or SAEs will be coded with MedDRA in its latest version.<br>The sponsor is responsible for implementing and maintaining quality assurance and quality control systems with written SOPs to ensure that trials are conducted and data are generated, documented (recorded), and reported in compliance with the protocol, GCP, and the applicable regulatory requirement(s).
Statistical methods	20a	The primary endpoint PFS will be analyzed with a Cox proportional hazards model, containing the randomized treatment as hypothesis variable and the stratification variable response status as a covariate. The test of the primary hypothesis (null hypothesis: equality of PFS rates) will be conducted within this model. The treatment effect will be described by the estimated hazard ratio from this model and will be presented with a two-sided 95% confidence interval. The null hypothesis will be rejected if the value 1 is not contained in the two-sided 95% confidence interval for the hazard ratio describing the relation between treatment groups. Additionally, the PFS rates will be estimated by the Kaplan-Meier method. The endpoint OS will be analyzed in the same way as described for PFS. The endpoint CR rate will be analyzed as the dependent variable of a logistic regression model with treatment assignment as independent variable. The endpoint response duration will be estimated with a Cox-regression model. Death without former progression will be analyzed as competing event. The regression models allow the inclusion of further potentially important prognostic factors. Details will be determined in a Statistical Analysis Plan (SAP) to be finalized before the analysis starts. For the evaluation of CR on day 60 after randomization, patients not completing therapy will be counted as non-responders. With respect to the endpoint QOL, the treatment groups will be compared descriptively according to the EORTC manual. All p-values from analyses of secondary endpoints will be interpreted in a descriptive sense
	20b	Subgroup analyses will be conducted based on response status (CR versus PR) as evaluated by contrast enhanced MRI at the time of randomization. Of note, response status is a stratification factor, thus the number of patients with either CR or PR between the two arms will very likely be balanced.<br>We hypothesize that particularly those patients with PR will benefit more from the more aggressive approach HDT-ASCT than patients with CR, because patients who do not respond completely after 4 cycles of conventional chemotherapy might be suffering from a lymphoma that is more resistant to conventional chemotherapy than the lymphomas in those patients who had already achieved a

complete response. Therefore, the response status can be considered as a surrogate for a certain lymphoma biology that tends to be more aggressive, something not yet well understood.

We will estimate these subgroup effects for PFS and OS using Cox regression analyses including the following variables in the models: treatment allocation and response status at randomization. An appropriate interaction term (treatment allocation*response status) will be added to the model. We will illustrate subgroup effects using forest plots and provide the P value for the interaction test. Whether the number of events (around 7 per variable) suffices to designate OS will have to be seen. If not, we will conduct the subgroup analyses only for PFS.

Based on the IELSG risk score, (24) we will evaluate the prognostic impact (PFS and OS) of the following baseline variables at the time of study inclusion before starting any chemotherapy: age (as continuous variable), performance score (ECOG 0-1 versus ECOG >1), liquor-protein elevation (yes versus no), involvement of deep-brain structures (yes versus no), and elevated serum LDH (yes versus no). We will use Cox regression analyses without variable selection procedures to explore these risk factors.

20c Further descriptive analyses will consider all patients included in the trial, i.e. data from start of induction therapy with Rituximab, MTX, Ara-C, Thiotepa will be analyzed. We will analyze PFS and OS starting at registration for all patients, and we will consider three groups (two treatment arms, not randomized).

Methods: Monitoring

Data monitoring 21a An independent Data Monitoring Committee (DMC) will be established. The DMC will consist of two medical scientists and one statistician with longstanding experience in clinical trials (see section Responsibility page 22). The DMC's function is to monitor the study's course and if necessary make recommendations to the steering committee for study discontinuation, modification or continuation the underlying principles for the DMC are the patients' ethical and safety aspects. It is the task of the DMC to examine whether the study's conduct is still ethically justifiable, whether security of the patients is ensured and whether the study's conduct is acceptable. The DMC will be informed about adherence to the protocol, patient recruitment, observed serious adverse events and deaths. The DMC will receive the corresponding reports (DSURs) at regular intervals. Recommendations on further continuation or modification of the study will be given to the steering committee. The composition and responsibilities of the DMC, the structure and procedures of its meetings, and its relationship to other key study team members (steering committee), will be laid down in a separate DMC charter.

	21b	An interim analysis will not be conducted in this trial. The sponsor/coordinating investigator is under obligation to monitor the progress of the clinical trial with regard to safety-relevant developments and, if necessary, initiate the premature termination of a treatment arm or the entire clinical trial. The sponsor/coordinating investigator will be supported in this responsibility by the DMC, if necessary.<br>A treatment arm or the entire clinical trial must be terminated prematurely if:<br>• the benefit-to-risk ratio for the patient changes markedly,<br>• the sponsor/coordinating investigator (German LKP) or the DMC considers that the termination of the trial is necessary,<br>• indications arise that the trial patients' safety is no longer guaranteed,<br>• the questions addressed in the trial can be clearly answered on the basis of results from another trial on the same subjects.
Harms	22	An adverse event (AE) is any untoward medical occurrence in a patient administered any dose of a pharmaceutical product and which does not necessarily have to have a causal relationship with the product's use. An AE can therefore be any unfavourable and unintended sign (including an abnormal laboratory finding), symptom, or disease temporally associated with the use of an investigational product, whether or not related to the product. Irrespective of any causal relationship, all AEs spontaneously reported by the patient or observed by the investigator will be continuously documented in the medical record and on the designated case report form (AE CRF page). All AEs must be described by diagnosis or, if an underlying diagnosis is not known, by symptoms or medically significant laboratory or instrumental abnormalities. The AEs will be documented as shown in the section below.<br>Symptoms or medically-significant laboratory or instrumental (e.g. electro-cardiographic) abnormalities concerning a pre-existing disease should not be considered an AE. However, occurrences of new symptoms or laboratory or instrumental abnormalities as well as worsening of pre-existing symptoms are considered AEs.<br>In order to monitor the conditions of the patients from the point in time when they receive the first dose of the investigational product, the investigator is requested to report any untoward clinical event on an AE-page of the CRF. Any untoward medical occurrence that occurs after the patient's follow-up period as defined in the protocol is not considered an AE. All AEs, no matter how intense, are to be followed up by the investigator in accordance with good clinical practice until resolved or judged no longer clinically relevant, or in case of a chronic condition, until it is fully characterized.

Adverse events must be documented in the CRF starting from the first administration of the study medication until day 60 after randomization. If a related SAE occurs after day 60 after randomization, it will be documented on the respective AE-page of the CRF. During induction and consolidation treatment and before beginning each chemotherapy cycle, the CRF-toxicity table must be thoroughly completed by the investigator. The investigator is to state the presence of a toxicity grade (CTCAE) in terms of several laboratory parameters and symptoms or diagnoses. If the symptom, diagnosis, or laboratory parameter is not stated on the toxicity table, an AE must be documented on the CRF AE page. If any of the seriousness criteria are fulfilled, an SAE must be reported. All SAEs that occur starting from the first administration of the study medication until day 60 after randomization will be documented in the CRF and on the provided SAE reporting form. This applies to events that are only documented on the toxicity table, that are related and not related to the study medications, and events of any CTCAE grade. After this time period, an SAE need only be reported to the sponsor and documented on the CRF if judged by the investigator to be related to at least one of investigational products. The SAE reporting form will be processed as described in the section below.

All SAEs must be reported by fax to the following address within 24 hours after knowledge by the investigator. In the event of the death of a patient, the investigator must submit all information to the competent ethics committee, the other ethics committee involved, and the competent authority and sponsor that is required for the fulfilment of his or her duties (note that personal data must be transmitted using the trial-specific patient identification number, i.e. in anonymized form). As this trial involves patients suffering from malignancies associated with significant mortality/morbidity, and because relapse/progression are trial endpoints (i.e. anticipated clinical outcomes) collected on the specific CRF pages and taking into consideration recommendations of the CIOMS working group VI concerning management of safety information from clinical trials, the investigator need not inform the sponsor about the events below, as they are not considered SAEs in this disease context: relapse or progression. A study patient’s relapse or progression will be documented on specific CRFs pages and should not be communicated to the Sponsor as SAEs. Nevertheless, the investigator must fax the CRF page designated for relapse documentation to the SAE Management Center CTU within 3 working days after knowledge.

The sponsor's expedited reporting requirements are particularly relevant to suspected unexpected serious adverse reactions (SUSARs). The definition is a combination of the definitions of serious adverse reaction and unexpected adverse reaction (an adverse reaction, the nature or severity of which is inconsistent with the applicable product information for the investigational medicinal

product). According to guideline CPMP/ICH/377/95 (ICHE2A), all events suspected by either the investigator or the sponsor as having a reasonable causal relationship to the administration of the investigational medicinal product qualify as suspected adverse reactions (SARs). The expression causal relationship means that there is evidence or argument to suggest a causal relationship between the event and the administration of the investigational medicinal product. According to guideline ENTR/CT 3, all cases of suspected unexpected serious adverse reactions for which a causal relationship with a investigational medicinal product (test or reference drug) is suspected by an investigator or the sponsor are patient to the expedited reporting requirements. The sponsor's reporting requirements are divided into expedited reporting and reporting that must be done annually or on request. The sponsor's expedited reporting requirements comprise the following:

all SUSARs must be reported within 15 days after knowledge (section 13, subsection 2 GCP-V), all SUSARs that are life-threatening or result in death must be reported within 7 days after knowledge, all circumstances requiring a review of the benefit/risk evaluation of the investigational medicinal product must be reported within 15 days after knowledge (e.g. expected serious adverse reaction with unexpected outcome, increased incidence of expected serious adverse reactions, SUSARs after the end of the patient's participation in the clinical trial, events in connection with the trial conduct or the development of the investigational medicinal product which may affect the safety of the trial patients).

In addition to the expedited reporting, the sponsor shall submit an annual report once a year or on request throughout the clinical trial period, according to section 13, subsection 6 GCP-V. According to ICH guideline E2F, this annual report should include the following points: a report on the safety of the trial patients in the clinical trial concerned, a list of all suspected serious adverse reactions (including all SUSARs) that occurred in the clinical trials concerned, a summary list of suspected serious adverse reactions (specified by organ system, treatment arm and type of adverse reaction) that occurred in the clinical trial concerned. To fulfill these requirements the investigator must fax the CRF page designated for premature treatment/study discontinuation to the SAE Management Center CTU within 3 working days after knowledge.

Any pregnancy that occurs during trial participation must be reported to the Sponsor on the SAE-reporting form within 24 hours of learning of its occurrence. The pregnancy should be followed up by the sponsor to determine outcome, including spontaneous or voluntary termination, details of birth, and the presence or absence of any birth defects, congenital abnormalities or maternal and newborn complications.

Auditing	23	According to the ICH-GCP guidelines, audits will be performed within a quality assurance system. Audits and/or inspections may be conducted by the sponsor, authority(ies) or an independent external party. The purpose of a sponsor's audit, which is independent of and separate from routine monitoring or quality control functions, should be to evaluate trial conduct and compliance with the protocol, SOPs, GCP, and the applicable regulatory requirements. All persons who conduct an audit undertake in writing to treat all data related to medical secrecy or which could reveal the patient's identity in absolute confidence, and to restrict the use of such data to the purposes agreed by the patient in writing. Proposed dates for sponsor's audit, characteristics of the selected patients and further information will be transmitted to the investigator by the CRA in a timely manner. The investigator will inform the CTU immediately of an inspection requested by a regulatory authority. The investigator is responsible for making source data/documents during audits or inspections available.

Ethics and dissemination

Research ethics approval	24	not applicable
Protocol amendments	25	Any change or addition to the protocol can only be made in a written protocol amendment that must be approved by sponsor, CA where required, and the IEC.<br>Only changes to the protocol that are required for patient safety may be implemented prior to IEC approval. Despite the need for approval for formal protocol amendments, the investigator is expected to take any immediate action required to ensure the safety of any patient enrolled in this trial, even if such action represents a protocol deviation. In such cases, the sponsor should be notified as soon as possible of this action; the IEC should be informed correspondingly.
Consent or assent	26a	Before enrollment in the clinical trial, the patient will be informed that participation in the clinical trial is voluntary and that he/she may withdraw from the clinical trial at any time without having to give reasons and without penalty or loss of benefits to which the patient is otherwise entitled. The treating physician will provide the patient with information about the treatment methods to be compared and the possible risks involved. At the same time, the nature, significance, implications, expected benefits and potential risks of the clinical trial and alternative treatments will be explained to the patient. During the informed consent discussion, the patient will also be informed about the insurance cover that exists and the insured's obligations. The patient will be given ample time and opportunity to obtain answers to any open questions. All questions relating to the clinical trial should be

answered to the satisfaction of the patient and/or his/her legal representative. In addition, the patient will be given a patient information sheet which contains all the important information in writing. By signing the consent form, the patient agrees to voluntarily participate in the clinical trial and declares his/her intention to comply with the requirements of the clinical trial and the investigator's instructions during the clinical trial. By signing the form, the patient also declares that he/she agrees to the recording of personal data, particularly medical data, for the trial, to their storage and codified ("pseudonymized") transmission to the sponsor, to the competent authority or the competent authority, and further agrees that authorized representatives of the sponsor University Medical Center Freiburg, who are bound to confidentiality, and national or foreign competent authorities may inspect his/her personal data, particularly medical data, which are held by the investigator. After signing, the patient will be given one copy of the signed and dated written consent form and any other written information to be provided to the patients. In the case of substantial amendments, the patient must be informed with an appropriate revised patient information/consent form. Changed trial procedures can only be carried out if they have been approved by the competent authority and the leading Ethics Committee, and if the patient has been appropriately informed and has given his/her written consent. Fertile men and women of child-bearing potential should be informed that taking the investigational product may involve unknown risks to the fetus if pregnancy were to occur during the trial and agree that in order to participate in the trial, they must adhere to the contraception requirement for the trial's duration. If there is any doubt that the patient may not reliably comply, they should not be entered in the trial.

26b For the additional BM aspirate, CSF and blood samples taken from patients participating in the Translational Research Program written informed consent will be obtained separately. For details please refer to the informed consent materials in the Appendix section.

Confidentiality

27 A confidential log of the names of all trial patients with the identification code* assigned to each patient at the time of enrollment in the clinical trial. With this list, each patient's identity can be revealed. The list must be kept confidential and must not leave the institution. It must remain at the trial center and must not be copied or otherwise passed on! Sponsor representatives, auditors and representatives of authorities must be allowed to inspect the list on request. Information about trial patients will be kept confidential and managed under the applicable laws and regulations. Those regulations require a signed patient authorization informing the patient of the following: what protected health information (PHI) will be

		collected from patients in this trial; who will have access to that information and why; who will use or disclose that information; right of trial patients to revoke their authorization for use of their PHI. In the event that a patient revokes authorization to collect or use PHI, the investigator, by regulation, retains the ability to use all information collected prior to the revocation of patient authorization. For patients who have revoked authorization to collect or use their PHI, attempts should be made to obtain permission to collect at their least vital status (i.e. that the patient is alive) at the end of their scheduled trial phase. The investigator must ensure anonymity of the patients; patients must not be identified by names in any documents submitted to the sponsor. Signed informed consent forms and patient enrollment log must be kept strictly confidential to enable patient identification at the site.
Declaration of interests	28	JF and GI receive speakers honoraries from Riemser Pharma GmbH. All other investigators declare that they have no competing interests.
Access to data	29	Sponsor and study chairman (coordinating principal investigator) is responsible for access to data set. This item is stipulated in details in the site contracts between sponsor, chairman (coordinating PI) and participating sites.
Ancillary and post-trial care	30	The treatment and care of patients after the end of study is conducted according to oncological and haematological standards in the respective sites.<br><br>Subject insurance (minimum: € 500,000 per subject) for all subjects participating in the clinical trial for compensation to those who suffer harm from trial participation.
Dissemination policy	31a	Upon trial completion, the results of this trial will be submitted for publication and/or posted in a publicly-accessible database of clinical trial results. Reporting guidelines will be taken into account (see www.equator-network.org), e.g. the CONSORT statement should be adhered to when drafting papers on the results of randomized studies.<br><br>Each publication of trial results will occur in mutual agreement between the principal investigator, any other investigators involved, and the CTU. All data collected in connection with the clinical trial will be treated in confidence by the coordinating investigator and all others involved in the trial until publication. Interim data and final results may only be published (orally or in writing) with agreement from the coordinating investigator and the CTU. This is essential for a thorough exchange of information between the afore mentioned parties and will ensure that the opinions of all parties involved have

been heard before publication. This agreement, which does not include any veto right or right of censorship for any of the parties involved, may not be refused without good reason.

31b Study chairman (coordinating principal investigator) is responsible for publication. Details with reference to publication rights are stipulated in the site contracts between sponsor, chairman (coordinating PI) and participating sites.

Any intended use of professional writers: not applicable

31c Plans, if any, for granting public access to the full protocol, participant-level dataset, and statistical code: not applicable

Appendices

Informed consent materials	32	Model consent form and other related documentation given to participants and authorised surrogates

Patientenetikett	Prüfstelle (Stempel der Einrichtung)

PATIENTENINFORMATION

Studientitel:
Randomisierte, kontrollierte, offene, multizentrische Phase III Studie mit 2 parallelen Armen - Hochdosis Chemotherapie und autologe Stammzelltransplantation oder konventionelle Chemotherapie zur Konsolidierung bei primären ZNS-Lymphomen – (Methotrexat, Ara-C, Thiotepa, Rituximab) – **MATRix / IELSG43**

Inhaltsverzeichnis

Seite	Kapitel	
**2**	**1**	Allgemeine Informationen zur Studie
**3**	**2**	Hintergrund und Bedeutung der Studie
**3**	**3**	Zielsetzung der Studie
**3**	**4**	Studienablauf und graphische Darstellung
**6**	**5**	Beschreibung der einzelnen Behandlungsabschnitte
**9**	**6**	Was muss ich während der Behandlung beachten?
**10**	**7**	Mangelndes Therapieansprechen
**10**	**8**	Risiken und Nebenwirkungen
**13**	**9**	Kosten
**13**	**10**	Wann darf ich nicht an dieser Studie teilnehmen?
**13**	**11**	Ausscheiden aus der Studie
**14**	**12**	Versicherungsschutz
**15**	**13**	Wo finde ich weitere Informationen zu dieser Studie?
**15**	**14**	Datenschutz
**16**	**15**	Probenaufbewahrung
**16**	**16**	Kontakt bei weiteren Fragen / eigene Notizen
**18**	**17**	Glossar (bezieht sich auf alle ***kursiv*** gedruckten Fachausdrücke)
**20**	**18**	Einwilligungserklärung / Datenschutzerklärung

Sehr geehrte Patientin,
sehr geehrter Patient,

die klinische Forschung arbeitet permanent an der Weiterentwicklung und Verbesserung von Therapien zur Behandlung unterschiedlichster Erkrankungen. Hierfür ist die Durchführung von klinischen Studien unerlässlich.

Diese klinischen Studien werden nach strengen rechtlichen und ethischen Anforderungen entwickelt und durchgeführt. Bevor die Studie starten kann, bedarf es einer zustimmenden Bewertung durch die zuständige Ethikkommission sowie einer Genehmigung durch die zuständigen Behörden.

Mit diesem Dokument möchten wir Sie um Ihre Einwilligung zur Teilnahme an einer klinischen Studie bitten.

Die folgenden Seiten sollen Sie über die Studie und die damit verbundenen wichtigen Inhalte informieren und Ihnen helfen, eine Entscheidung über Ihre Teilnahme zu treffen. Das Dokument dient als Grundlage für das anschließende Gespräch mit Ihrem Arzt. Hier können Sie Fragen stellen und Dinge ansprechen, die gegebenenfalls in diesem Dokument nicht berücksichtigt wurden. Nach dem Gespräch mit dem Prüfarzt werden Sie ausreichend Bedenkzeit erhalten, um über eine Teilnahme zu entscheiden.

Ihre Studienteilnahme ist freiwillig und bedarf Ihrer schriftlichen Einwilligung.

Das Behandlungsprotokoll dieser Studie entspricht dem neuesten Wissensstand über die Therapie Ihrer Erkrankung. Sollten nach Therapiebeginn neue Erkenntnisse bekannt werden, die Ihre Bereitschaft zur Teilnahme beeinflussen könnten, werden Sie umgehend informiert.

Sollten Sie nicht an der Studie teilnehmen oder zu einem späteren Zeitpunkt ausscheiden wollen, erwachsen Ihnen daraus keinerlei Nachteile.

1 ALLGEMEINE INFORMATIONEN ZUR STUDIE

Die geplante klinische Studie beschäftigt sich mit dem Vergleich zweier unterschiedlicher Behandlungsmöglichkeiten bei bösartigen primären Lymphomen des zentralen Nervensystems. Die in dieser Studie eingesetzten Medikamente werden bereits seit vielen Jahren für die Behandlung dieser Krankheit verwendet.

An dieser Studie beteiligen sich deutschlandweit 30 Krankenhäuser und Universitätskliniken sowie mindestens 15 Kliniken in Italien, England und der Dänemark. Insgesamt werden 250 Patienten in die Studie aufgenommen. Die Behandlungsdauer beträgt pro Patient ca. 20 Wochen. Bereits während der Chemotherapie (nämlich nach zwei und vier Therapiezyklen) sowie ca. einen Monat nach Therapieabschluss findet eine Erfolgskontrolle statt. Weitere Kontrolluntersuchungen werden in den ersten beiden Jahren alle drei Monate durchgeführt. In den darauf folgenden drei Jahren erfolgen die Kontrolluntersuchungen alle sechs Monate, ab dem sechsten Jahr einmal jährlich.

2 HINTERGRUND UND BEDEUTUNG DER STUDIE

Bei Ihnen ist eine bösartige Erkrankung des lymphatischen Systems im Bereich des Gehirns und/oder des Rückenmarks festgestellt worden – ein sogenanntes malignes Non-Hodgkin-Lymphom (NHL) des Zentralnervensystems (ZNS). Dabei handelt es sich um eine Ansammlung bösartiger Immunzellen (*Lymphozyten*), die unbehandelt innerhalb kurzer Zeit zum Tode führen können.

Früher galt die Bestrahlung des Gehirns bei Patienten mit primären ZNS-Lymphomen als Standardbehandlung. Bei ausschließlicher Bestrahlung ist jedoch bei der Mehrzahl der Patienten nach Ende dieser Behandlung mit einem erneuten Auftreten der Erkrankung zu rechnen.

Heute erhalten die Patienten zur Behandlung dieser Krankheit in der Regel eine kombinierte Behandlung aus mehreren *Chemotherapeutika*, gegebenenfalls in Kombination mit einer Strahlentherapie.
Verschiedene *Chemotherapeutika* werden bereits seit vielen Jahren als Standardbehandlung bei Lymphomerkrankungen außerhalb des zentralen Nervensystems eingesetzt. Inzwischen hat sich gezeigt, dass diese Substanzen auch bei der Behandlung von ZNS-Lymphomen sehr gut wirksam und sehr gut verträglich sind.

Ziel der Ihnen in der Studie vorgeschlagenen Behandlungsmethoden ist es, den Tumor mit den speziell für primäre Lymphome des zentralen Nervensystems ausgewählten *Chemotherapien* komplett zu vernichten. Diese Behandlung wird in mehreren Abschnitten durchgeführt.
Allen eingesetzten Substanzen ist dabei gemeinsam, dass sie insbesondere im Bereich des zentralen Nervensystems sehr gut wirksam sind.

3 ZIELSETZUNG DER STUDIE

Ziel dieser Studie ist es, die Wirksamkeit und Verträglichkeit zweier Behandlungen zu untersuchen und diese miteinander zu vergleichen, um definitiven Aufschluss darüber zu erhalten, welche der beiden Therapien die bessere ist.

Aussagen zum Nutzen verschiedener Behandlungsmöglichkeiten stützen sich grundsätzlich auf einen Vergleich dieser Möglichkeiten. Um einen fairen Vergleich der verschiedenen Behandlungsmöglichkeiten zu gewährleisten, sind gleiche Ausgangs- und Rahmenbedingungen erforderlich. Dies lässt sich nur dadurch erreichen, dass bei jedem Patienten und jeder Patientin per Losverfahren entschieden wird, welche der zu vergleichenden Behandlungen er oder sie erhalten wird.

4 STUDIENABLAUF UND GRAPHISCHE DARSTELLUNG

4.1 Alle Patienten erhalten zunächst die gleiche Behandlung. Sie besteht aus der sogenannten *Induktionschemotherapie* (=einleitende Therapie), bei der den Patienten über einen Zeitraum von jeweils zwei mal drei Wochen die

Medikamente *Rituximab, Methotrexat (MTX), Cytarabin (Ara-C) und Thiotepa* verabreicht werden.
Nach drei Wochen erfolgt eine erste Erfolgskontrolle, bei der das Ansprechen des Tumors auf die Behandlung mittels eines bildgebenden Verfahrens (in der Regel *Kernspintomographie, ggf. Computertomographie*) überprüft wird.
Bei gutem Ansprechen des Patienten auf die Behandlung wird diese wiederum über einen Zeitraum von zwei mal drei Wochen wiederholt mit einer anschließenden zweiten Erfolgskontrolle (*Kernspintomographie, ggf. Computertomographie*) am Ende.

4.2 Jetzt ist der Zeitpunkt gekommen, ab dem die Patienten per Losverfahren in die beiden Behandlungsgruppen Arm A und Arm B aufgeteilt werden. Dieses vorher festgelegte Zufallsverfahren, vergleichbar mit dem Werfen einer Münze, nennt man *Randomisierung*.

Hier die beiden Behandlungen, welche dem jeweiligen Arm zugeordnet werden:

Arm A	Patienten erhalten eine *konventionelle Chemotherapie* mit fünf Substanzen (*R-DeVIC-Protokoll*)
Arm B	Patienten erhalten eine Hochdosis-Chemotherapie mit Transplantation ihrer eigenen gewonnenen Blut*stammzellen* (autologe Stammzelltransplantation = HDT-ASZT)

W I C H T I G :
Für beide Behandlungsalternativen ist die Wirksamkeit bei ZNS-Lymphomen belegt. Die Studie soll definitiven Aufschluss darüber geben, welche der Kombinationen die bessere ist.

<u>Einteilung der Patienten in Gruppen</u>

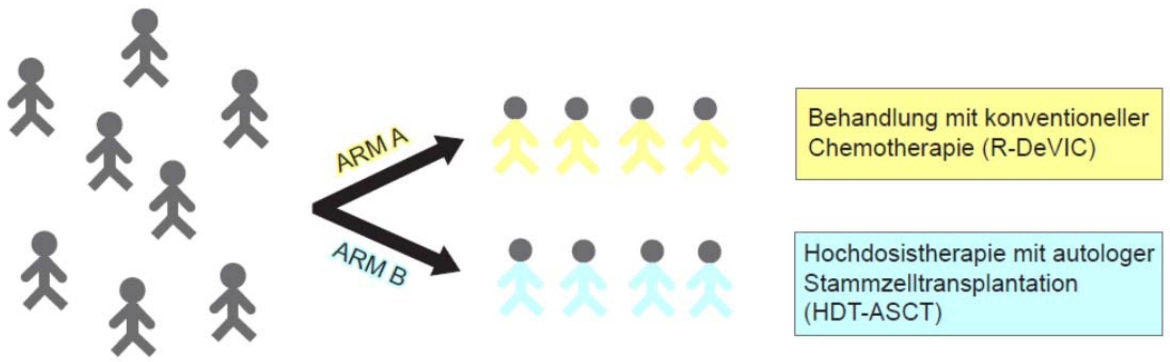

Graphische Darstellung des gesamten Studienablaufs

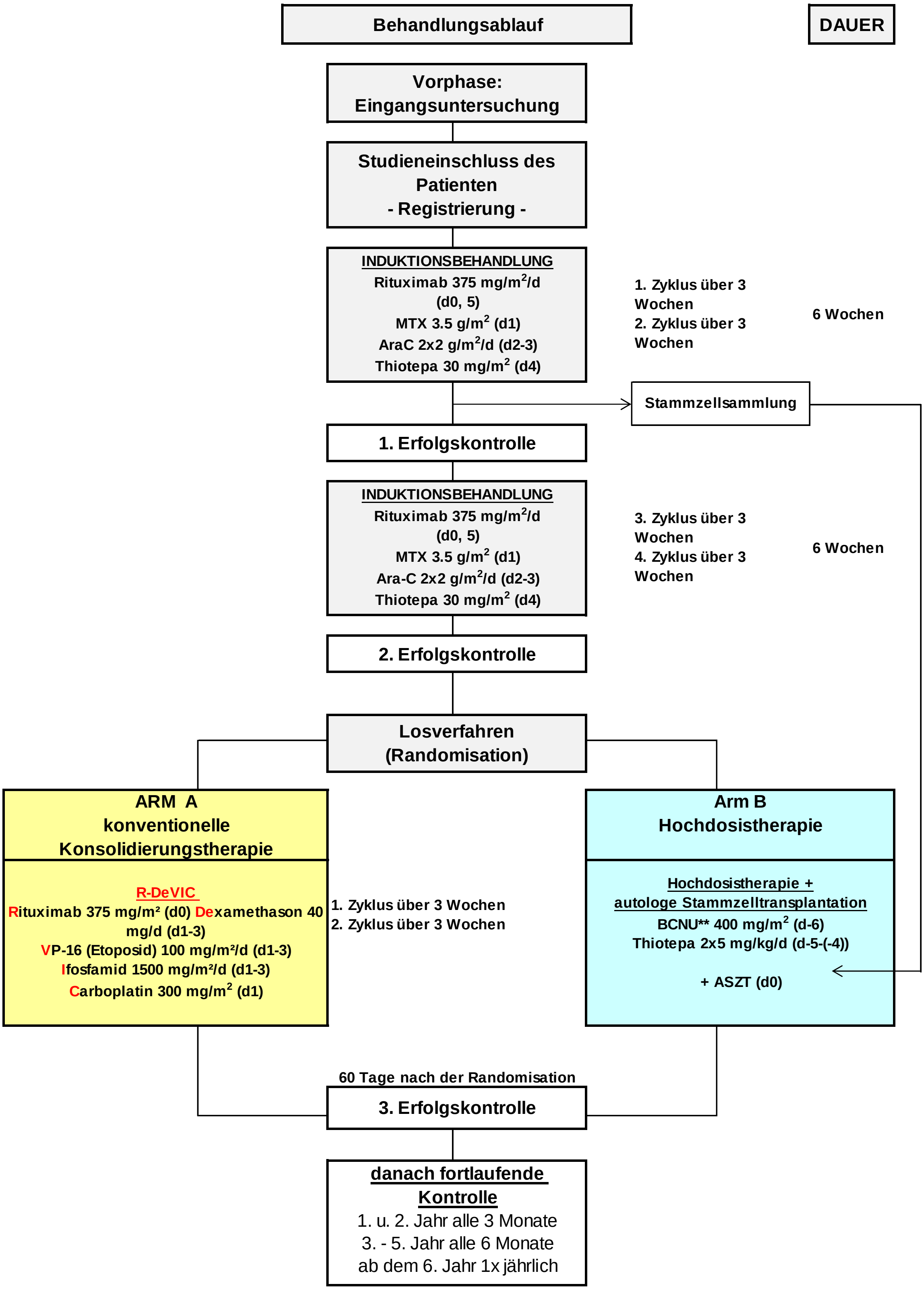

5 BESCHREIBUNG DER EINZELNEN BEHANDLUNGSABSCHNITTE

Vorphase

Bevor Sie in die Studie aufgenommen werden können, wird Ihr Arzt alle Ein- und Ausschlusskriterien als Voraussetzung für Ihre Teilnahme überprüfen.

Bei Aufnahme in diese klinische Prüfung wird die Vorgeschichte Ihrer Krankheit erhoben, und Sie werden einer umfassenden ärztlichen Untersuchung unterzogen. Die Möglichkeit Ihrer weiteren Teilnahme an dieser klinischen Prüfung wird von den Ergebnissen dieser Voruntersuchung abhängen. Sind alle Einschlussvoraussetzungen erfüllt, erfolgt die Registrierung.

Bei dieser klinischen Prüfung sollen zusätzlich zu den Routine- und Standarduntersuchungen folgende studienbedingte Maßnahmen durchgeführt werden:

Im Verlauf der Voruntersuchungen werden Tests durchgeführt, die dazu dienen, Ihre Denk- und Merkfähigkeit, Ihre Konzentrationsfähigkeit sowie Ihre Aufmerksamkeit aktuell einschätzen zu können. Dieser Test heißt "Neuropsychologische Testbatterie" und dauert in der Regel ungefähr 30 Minuten.

Zusätzlich bekommen Sie einen dreiseitigen, *pseudonymisierten* Fragebogen ausgehändigt, den Sie selbständig ausfüllen und in einem verschlossenen Umschlag zurückgeben. Hierbei wird Ihre persönliche Einschätzung Ihrer Lebensqualität erfasst.

Vor Beginn der eigentlichen *Chemotherapie* werden Sie gegebenenfalls für einige Tage Cortison in Form des Medikaments *Dexamethason* als Tabletten oder als *intravenöse Infusion* erhalten. Ziel ist es, dadurch die Schwellung des Gehirns im Bereich des Lymphoms zu verkleinern. Diese Behandlung wird nach Beginn des ersten Behandlungsabschnittes, der mit der Gabe des Studienmedikamentes *Rituximab* startet, durch schrittweise Verringerung der Dosis langsam abgesetzt.

Des Weiteren wird vor Beginn der Chemotherapie eine *Kernspintomographie* des Gehirns zur Überprüfung der Ausbreitung des Lymphoms durchgeführt. Dabei wird ein *Kontrastmittel* verwendet. Falls bei Ihnen z.B. wegen eines Herzschrittmachers oder aufgrund anderer Gegenanzeigen keine Kernspintomographie möglich ist, wird eine *Computertomographie (CT)* mit *Kontrastmittel* erfolgen.

***Induktionschemotherapie* (einleitende Therapie)**

Zunächst erhalten alle Patienten eine *Induktionschemotherapie* mit den Studienmedikamenten *Rituximab, MTX (Methotrexat), Ara-C und Thiotepa*. Die *Chemotherapie* findet immer während eines Klinikaufenthaltes (ca. sechs bis acht Tage) statt. Die Aufenthaltsdauer wird von Ihrem behandelnden Arzt bestimmt und kann je nach Ihrem Befinden oder organisatorischen Gegebenheiten auch länger dauern.

Unmittelbar vor und nach der *Chemotherapie* erhalten alle Patienten das Medikament *Rituximab*. Dabei handelt es sich um eine Form der *Immuntherapie*, d.h. eine über das körpereigene Abwehrsystem wirkende Behandlung, die sich gezielt gegen die bösartigen Lymphomzellen richtet. Diese Therapie wird in den letzten Jahren zunehmend und mit großem Erfolg bei Patienten mit bösartigen Lymphomen in Kombination mit einer *Chemotherapie* eingesetzt. Diese Therapie wird jeweils am Tag vor und nach der Chemotherapie (Tag 0 und 5) verabreicht.

An Tag 1 erfolgt eine Behandlung mit einer dreistündigen intravenösen Infusion mit dem *Chemotherapeutikum MTX* über einen *zentralvenösen Katheter*, der in die Halsvene gelegt wird. *Ara-C* wird am zweiten und dritten Tag insgesamt vier Mal als einstündige *Infusion* in einem Abstand von jeweils 12 Stunden verabreicht.

MTX und *Ara-C* sind seit mehreren Jahrzehnten gebräuchliche Medikamente, die breiten Einsatz in der Behandlung von Leukämien und Lymphomen finden und bei

primär zerebralen Lymphomen als besonders wirksam gelten. Etwa 24 Stunden nach der jeweiligen *MTX-Infusion* wird Ihnen ein weiteres Medikament (*Leukovorin*) zur Verringerung der Nebenwirkungen verabreicht. Dieses erhalten Sie alle sechs Stunden, bis kein *MTX* mehr in Ihrem Blut nachgewiesen werden kann. Am Folgetag der Behandlung mit *Cytarabin* erhalten Sie *Thiotepa* in Form einer halbstündigen *Infusion*.

Die Gabe dieser Substanzen wird in identischer Form nach drei Wochen als zweiter Zyklus wiederholt.

Nach Beendigung des zweiten *Zyklus* erfolgt die erste Erfolgskontrolle. Dazu wird eine mit *Kontrastmittel* durchgeführte Kernspintomographieuntersuchung (bzw. ggf. alternativ *Computertomographie)* des Gehirns durchgeführt.

Sollte sich das Lymphom nach den ersten beiden *Zyklen* vergrößert haben, erfolgt die weitere Behandlung außerhalb der Studie.

Sollte sich das Lymphom nach den ersten beiden *Zyklen* nicht vergrößert haben, folgen zwei weitere *Zyklen* (dritter und vierter *Zyklus*) mit den jeweils bereits zuvor angewendeten Medikamenten.

Anschließend wird erneut eine *Kernspintomographieuntersuchung (bzw.* alternativ eine *Computertomographie)* als zweite Erfolgskontrolle durchgeführt.

***Stammzell*sammlung**

Nach dem zweiten *Zyklus* (und bei Bedarf erneut nach dem dritten *Zyklus*) erfolgt die Ausschwemmung (=Mobilisierungsphase) von blutbildenden *Stammzellen*. Dies ist die Voraussetzung, um eine Hochdosis-Chemotherapie durchführen zu können. Blutstammzellen sind Zellen, die im Knochenmark enthalten sind und deren Rückgabe nach intensiver Therapie sowohl die gesamte Blutbildung als auch die Zellen des Immunsystems wieder herstellen können. Die Sammlung der Blutstammzellen findet bei allen Patienten statt, die an der Studie teilnehmen. Allerdings werden die gesammelten und tiefgefrorenen Zellen nur denjenigen Patienten wieder zugeführt, die für eine Hochdosistherapie, also Arm B, ausgelost wurden oder im Verlauf aufgrund eines Wiederauftretens des Lymphoms nach Studienende eine Hochdosis-Chemotherapie benötigen.

Zur Gewinnung dieser Zellen werden Sie 5 Tage nach Beendigung der Chemotherapie den Wachstumsfaktor *G-CSF* 1x täglich als *Injektion* unter die Haut gespritzt bekommen. Dies unterstützt, zusammen mit der zuvor durchgeführten Chemotherapie, die Ausschwemmung der *Stammzellen* aus dem Knochenmark ins Blut, wo sie mittels Blutstammzellsammlung (*Leukapherese*) gesammelt und für den späteren Einsatz tiefgefroren werden können.

Bei dieser *Leukapherese* werden die Blutstammzellen mittels eines speziellen Verfahrens aus Ihrem Blut entnommen. Die weißen und roten Blutkörperchen sowie die Blutplättchen werden Ihnen während der *Leukapherese* wieder zurückgegeben. Die *Stammzellen* werden bis zum Abschluss der Hochdosis-Chemotherapie eingefroren und einige Tage danach im Rahmen der Stammzelltransplantation mittels *Transfusion* in Ihren Körper zurückgeführt.

Erfolgskontrolle

Vor jedem Therapieabschnitt sowie nach der Stammzelltransplantation werden zur Überprüfung der Wirksamkeit der Behandlung *Kernspintomographien* bzw.

Computertomographien des Schädels durchgeführt; diese Untersuchungen erfolgen jeweils mit *Kontrastmittel*. Sollte sich dabei eine Größenzunahme des Lymphoms oder ein ungenügendes Therapieansprechen zeigen, muss die Therapie innerhalb der Studie abgebrochen werden und die weitere Behandlung mit alternativen Therapiemöglichkeiten außerhalb der Studie erfolgen.

Losverfahren (*Randomisierung*)

Nach der Erfolgskontrolle im Anschluss an den vierten *Zyklus* der Induktionschemotherapie findet die *Randomisierung* statt.

Dabei wird per Losverfahren entschieden, ob im weiteren Verlauf eine *konventionelle Chemotherapie* mit vier weiteren Substanzen (**Arm A)** oder eine Hochdosis- *Chemotherapie* mit *Rückgabe* Ihrer zuvor gewonnenen Blutstammzellen (**Arm B)** erfolgt.

Arm A

Konventionelle Chemotherapie (R-DeVIC)

Chemotherapie mit *Rituximab, Dexamethason, VP-16 (Etoposid), Ifosfamid, Carboplatin*:

Drei Wochen nach dem vierten *Zyklus* der Induktionschemotherapie erfolgt die Konsolidierungstherapie, d.h. die Verabreichung der zwei jeweils dreiwöchigen Behandlungszyklen in Arm A mit den Substanzen *Rituximab, Dexamethason, Etoposid, Ifosfamid und Carboplatin - R-DeVIC.*

Diese Therapie wird im Rahmen eines *stationären* Aufenthaltes an vier aufeinanderfolgenden Tagen verabreicht. Insgesamt müssen Sie pro *Zyklus* mit einem mindestens fünf- bis sechstägigen Klinikaufenthalt rechnen. Die Aufenthaltsdauer wird von Ihrem behandelnden Arzt bestimmt und kann je nach Ihrem Befinden oder organisatorischen Gegebenheiten auch länger dauern.

Rituximab erhalten Sie wie bei der Induktionstherapie am Tag vor der Chemotherapie (Tag 0). An Tag 1 werden alle vier weiteren Medikamente (*Dexamethason, Carboplatin, Ifosfamid und Etoposid*) verabreicht. Die Verabreichung von *Dexamethason* erfolgt über eine 15-minütige *Infusion.* Alternativ kann das Medikament auch in Form einer Tablette mit dem identischen Wirkstoff eingenommen werden. Im Abstand von einer Stunde wird Ihnen über eine einstündige Infusion Carboplatin verabreicht. Dieser Behandlung folgt eine zweistündige Infusion von *Ifosfamid.* Anschließend erhalten Sie *Etoposid*, welches ebenfalls über zwei Stunden intravenös verabreicht wird. An den beiden darauf folgenden Therapietagen wird nur *Dexamethason*, *Etoposid* und *Ifosfamid* entsprechend dem beschriebenen Schema verabreicht.

Diese gesamte Therapie wird nach drei Wochen wiederholt.

Arm B

Hochdosis-Chemotherapie mit autologer Stammzelltransplantation (HDT-ASZT):

Drei Wochen nach dem vierten *Zyklus* der *Induktions-Chemotherapie* erfolgt die Durchführung der Hochdosis-Chemotherapie mit *autologer* Stammzelltransplantation für die Patienten in Arm B, für die Sie mit einem ca. drei- bis fünfwöchigen stationären Aufenthalt rechnen müssen. Die Behandlung besteht aus einer Infusion zweier *Chemotherapeutika*: zunächst eine einstündige *intravenöse* Verabreichung von *Carmustin (BCNU) oder* alternativ *Busulfan* an einem Tag, gefolgt von vier jeweils zweistündigen Infusionen mit Thiotepa an den darauf

folgenden zwei Tagen (jeweils im Abstand von 12 Stunden). Diese müssen über einen *zentralvenösen Katheter*, der Ihnen vor Therapiebeginn in eine Halsvene gelegt wird, verabreicht werden. Um die Regeneration der Blutbildung zu fördern, werden vier Tage nach Beendigung der *Chemotherapie*, d.h. wenn die *Zytostatika* aus dem Körper ausgeschieden sind, die gesammelten *Stammzellen* aufgetaut und Ihrem Körper über den *zentralvenösen* Katheter zurückgegeben (Transplantationsphase der Stammzelltransplantation). Die *Stammzellen* siedeln sich im Knochenmark an und beginnen mit der Blutbildung, so dass ca. 10 bis 14 Tage nach der Rückgabe der *Stammzellen* das Blutbild soweit wiederhergestellt ist, dass keine schwere Infektions- und Blutungsgefahr mehr bestehen sollte.

Fortlaufende Nachsorgeuntersuchungen für alle Patienten aus beiden Gruppen

Nach Abschluss der gesamten Behandlung erfolgt die weitere Nachsorge durch Ihre behandelnde Klinik. Dazu sind in den ersten beiden Jahren dreimonatige Kontrollen notwendig. Die Nachsorgeuntersuchungen umfassen eine körperliche Untersuchung, eine *Kernspintomographie*-Untersuchung des Schädels (alternativ CT-Schädel), Routineblutuntersuchungen sowie jährlich das bereits beschriebene neurologische Assessment inklusive Fragebogen zur Lebensqualität.

Nach dem zweiten Jahr der Nachbeobachtung ist die Studie offiziell für Sie beendet. Unabhängig von der Studie findet danach jedoch weiterhin Ihre Tumornachsorge statt: vom dritten bis zum fünften Jahr sind halbjährliche Kontrollen empfohlen. Anschließend sollten einmal jährlich die gleichen Untersuchungen durchgeführt werden wie die oben genannten Kontrolluntersuchungen innerhalb der ersten beiden Jahre der Nachsorge. Wir bitten Sie um die Einwilligung, Ihre in diesem Zusammenhang erhobenen Daten ebenfalls in die Auswertung aufnehmen zu dürfen.

Im Rahmen der Nachsorge kann es sein, dass Sie telefonisch durch das Studienpersonal Ihrer Klinik kontaktiert werden, um die Dokumentation Ihrer Daten zu vervollständigen.

6 WAS MUSS ICH WÄHREND DER BEHANDLUNG BEACHTEN?

Meldung an Prüfarzt beim Auftreten von Symptomen, Begleiterscheinungen und auch bei Verletzungen

Da Sie zwischen den einzelnen Therapiezyklen nach Hause entlassen werden, ist Ihre Kooperation für das Gelingen der Therapie von entscheidender Bedeutung. Neben regelmäßigen Kontrollen des Blutbildes bei Ihrem Hausarzt sollten Sie sich bei allen Unregelmäßigkeiten, aber im Besonderen bei Fieber (> 38°C) oder Hinweisen für eine Infektion wie Brennen beim Wasserlassen oder Abhusten verfärbten Auswurfs sowie Blutungszeichen sofort mit Ihrem behandelnden Arzt in Verbindung setzen.

Zusätzliche Medikamente (auch rezeptfreie), von denen der Prüfarzt noch nichts weiß, sollten Sie – außer bei Notfällen – nur nach Rücksprache mit Ihrem Prüfarzt einnehmen. Wenn Sie von anderen Ärzten behandelt werden, sollten Sie diese über

Ihre Teilnahme an der klinischen Prüfung informieren. Auch Ihr Prüfarzt muss über jede medizinische Behandlung, die Sie durch einen anderen Arzt während der klinischen Prüfung erhalten, informiert werden. Sie sollten ohne ärztliche Beratung Medikationen weder wechseln noch absetzen.

7 MANGELNDES THERAPIEANSPRECHEN

Nach Beendigung der Therapie werden zu bestimmten Zeitpunkten in der oben bereits beschriebenen Weise weitere *Kernspintomographien* (bzw. *Computertomographie*n) des Gehirns zur Überprüfung des Therapieerfolges und des weiteren Verlaufs durchgeführt.

Eine vereinfachte Darstellung des gesamten Behandlungsablaufes finden Sie im Behandlungsplan auf Seite 5 dieser Patienteninformation.

Durch diese schrittweise Behandlung strebt man einen Rückgang des Tumors an mit dem Ziel, ein dauerhaftes Verschwinden aller Tumorzellen, d.h. eine Heilung, zu erreichen.

Es ist jedoch nicht sicher, dass alle Tumorzellen tatsächlich verschwinden werden. Es ist möglich, dass der Tumor nicht oder nicht ausreichend auf die Therapie anspricht oder zu einem späteren Zeitpunkt wieder auftritt, nachdem er im Verlauf der Behandlung mittels Kernspintomographie oder Computertomographie nicht mehr festgestellt werden konnte.

Ihr Arzt wird in diesem Fall alternative Behandlungsmöglichkeiten mit Ihnen besprechen.

8 RISIKEN UND NEBENWIRKUNGEN

8.1 Nebenwirkungen der Prüfmedikamente

Vor Beginn der Chemotherapie werden Sie durch Ihren behandelnden Arzt nochmals ausführlich mündlich sowie schriftlich über die Risiken und Nebenwirkungen der jeweiligen Therapie aufgeklärt. Bei geschäftsunfähigen Patienten erfolgt die Aufklärung des gesetzlichen Vertreters oder des Bevollmächtigten.

Zu allen *Chemotherapie-Infusionen* erhalten Sie begleitend Medikamente, die mögliche Nebenwirkungen unterdrücken bzw. vermeiden sollen. Dennoch können - in der Regel vorübergehend - folgende Nebenwirkungen auftreten:

Regelhaft kommt es zu Veränderungen des Blutbildes mit Abnahme der weißen und roten Blutkörperchen sowie der Blutplättchen mit einhergehender Blutungsneigung bzw. Infektanfälligkeit. Des Weiteren kann es zu Übelkeit mit Erbrechen, Entzündungen der Schleimhäute im Mund und des gesamten Magen-Darm-Traktes, Durchfällen sowie zu Einschränkungen der Nieren-, Leber- und Lungenfunktion kommen, akut sowie chronisch. Selten können Veränderungen des Gefühls in Händen und Füßen sowie zentralnervöse Störungen, d.h. Störungen der Gehirn- und Rückenmarkfunktion (z.B. Denk- und Bewegungsstörungen) auftreten. In äußerst seltenen Fällen kann es zu allergischen Reaktionen bis hin zum Schock mit lebensbedrohlichen Zuständen kommen. Lokale Hautreizungen bis zur akuten Entzündung können bei paravenöser Injektion (=unbeabsichtigte Injektion außerhalb

der Vene) auftreten. Bei Rötung/Schmerzen an der Injektionsstelle benachrichtigen Sie bitte unverzüglich Ihren behandelnden Arzt.

Neben den oben genannten Nebenwirkungen ist ein häufig vorübergehendes Ausbleiben der Regelblutungen bei Frauen wahrscheinlich, eine bleibende Unfruchtbarkeit ist möglich. Für Männer besteht ebenfalls das Risiko der Unfruchtbarkeit. Bei bestehendem Kinderwunsch ist grundsätzlich eine Spermakonservierung bzw. eine Konservierung von Eizellen vor Beginn der Behandlung möglich. Ihr betreuender Arzt kann Ihnen hierzu weitere Informationen geben. Ab der schriftlichen Einwilligungerklärung während der gesamten Studie und bis zwei Jahre nach der Studientherapie sollten wegen der möglichen Gefahr der Erbgutschädigung keine Kinder gezeugt werden. Sollte trotz Empfängnisverhütung eine Schwangerschaft eintreten, muss dies Ihren behandelnden Ärzten unverzüglich mitgeteilt werden.

Grundsätzlich besteht nach einer Chemotherapie ein erhöhtes Risiko einer weiteren Tumorerkrankung im Verlauf von Jahren oder Jahrzehnten. Des Weiteren besteht die Möglichkeit des Wiederkehrens der jetzigen Erkrankung nach der Behandlung. Die Höhe dieses Risikos hängt stark von der Art und der Ausbreitung der Erkrankung ab.

8.2 Nebenwirkungen der Leukapherese (=Stammzellgewinnung)

Für das mehrstündige Verfahren der Zellgewinnung benötigt man zwei *Kanülen* in beiden Armbeugevenen, wie sie auch bei einer Blutspende verwendet werden. Nur bei ungünstigen Venenverhältnissen wird ein *zentralvenöser Katheter* benötigt, der am Hals unter örtlicher Betäubung gelegt wird. Abgesehen von diesen technischen Erfordernissen ist die *Leukapherese* in der Regel nebenwirkungsarm. Möglicherweise kann es während dieser Behandlung zu Kribbeln und Müdigkeit kommen; Nebenwirkungen, die durch Kalziumgaben gut beherrschbar sind. Darüber hinaus können durch die Kanülen oder den zentralen Venenkatheter Blutungen, Verletzung von Nerven mit möglicherweise dauerhaften Missempfindungen oder Taubheitsgefühl und Infektionen auftreten. Letztere werden gegebenenfalls mit Antibiotika behandelt. Lediglich Blutungen in Form von Blutergüssen sind relativ häufig. Alle weiteren Komplikationen durch die Kanülen bzw. den Katheter sind sehr selten. Sie werden vor der Stammzellsammlung darüber nochmals gesondert aufgeklärt und gebeten, hierzu Ihre schriftliche Einwilligung zu geben.

8.3 Nebenwirkungen der Hochdosis-Chemotherapie und der *autologen* Stammzelltransplantation

Die Hochdosis-Chemotherapie mit anschließender *autologer* Stammzell-transplantation ist ein Routineverfahren, das jährlich tausendfach bei Patienten mit verschiedenen bösartigen Erkrankungen wie Lymphomen außerhalb des ZNS und anderen Krebserkrankungen durchgeführt wird. Auch beim Lymphombefall des Gehirns wird diese Therapie bereits erfolgreich eingesetzt. Sowohl durch verschiedene unterstützende Maßnahmen im Verlauf der Therapie als auch durch die umfangreiche Erfahrung Ihrer behandelnden Ärzte mit diesem Verfahren ist eine größtmögliche Therapiesicherheit gegeben.

Prinzipiell besteht jedoch auch trotz aller Vorsichtsmaßnahmen das Risiko lebensbedrohlicher Komplikationen mit möglichem tödlichem Ausgang. Vornehmlich ist hier das Risiko von schwerer Infektion oder Blutung zu nennen. Dieses tödliche

Risiko liegt entsprechend internationaler Studien mit großen Patientenzahlen unter 5%.

Prinzipiell können im Rahmen der Hochdosis-Chemotherapie die gleichen Nebenwirkungen wie bei einer *konventionellen Chemotherapie* auftreten. Erfahrungsgemäß ist jedoch die Störung der Blutbildung so ausgeprägt, dass ungefähr fünf bis 12 Tage nach der Therapie eine deutlich erhöhte *Infekt*- und Blutungsneigung besteht, so dass Sie in diesen Tagen bei einem Infekthinweis (Fieber, Schüttelfrost, Erhöhung der Infektwerte im Blut) Antibiotika verabreicht bekommen. Im Rahmen der Therapie wird es zu einer verminderten Bildung der roten Blutkörperchen und der Blutplättchen kommen. Sollte die Verringerung dieser Blutzellen sehr ausgeprägt sein, kann die Transfusion von Blutkonserven und ggf. Blutplättchenkonzentraten notwendig werden. Die Blutprodukte werden von ausgewählten Spendern zur Verfügung gestellt und sind dahingehend untersucht, dass das Risiko einer Infektion weitgehend vermieden wird, jedoch aus technischen Gründen niemals komplett auszuschließen ist. Im Falle der Notwendigkeit von *Transfusionen* erfolgt eine separate Aufklärung.

Um die Regeneration der Blutbildung zu fördern, werden vier Tage nach Beendigung der Chemotherapie, d.h. wenn die *Zytostatika* aus dem Körper ausgeschieden sind, die gesammelten Stammzellen aufgetaut und Ihrem Körper über den zentralvenösen Venenzugang zurückgegeben (Transplantationsphase der Stammzelltransplantation). Die Stammzellen siedeln sich im Knochenmark an und beginnen mit der Blutbildung, so dass ca. 10-14 Tage nach der Rückgabe der Stammzellen das Blutbild soweit wiederhergestellt ist, dass keine akute Infektions- und Blutungsgefahr mehr bestehen sollte. Bei ausreichender Stammzellsammlung und -verabreichung ist das Risiko, dass die Stammzellen nicht anwachsen, sehr gering. Die Wahrscheinlichkeit liegt nach aktuellem Kenntnisstand bei unter 2%.

Daneben können die Veränderungen der Schleimhäute vorübergehend so ausgeprägt sein, dass eine Ernährung nur über den zentralvenösen Katheter möglich ist, ebenso kann es notwendig sein, vorübergehend intravenös Schmerzmittel zu verabreichen.

Nach Normalisierung der Blutwerte können Sie in Abhängigkeit von Ihrem körperlichen Zustand in die ambulante Weiterbetreuung entlassen werden. Erfahrungsgemäß erfolgt unter normalen Umständen eine rasche Erholung nach der Therapie. In den ersten Wochen verspüren Sie häufig noch eine leichte Einschränkung Ihrer Leistungsfähigkeit. Wichtig ist es, dass Sie sich beim Auftreten von Fieber oder anderen Anzeichen für Infekte sofort bei Ihrem behandelnden Arzt melden. Grundsätzlich sollte eine stationäre Rehabilitation erfolgen, diesbezüglich wenden Sie sich an Ihr Behandlungsteam und den Sozialdienst Ihres Krankenhauses.

9 KOSTEN

Im Rahmen der Teilnahme an dieser klinischen Studie entstehen Ihnen gegenüber der üblichen Therapie keine zusätzlichen Kosten.

10 WANN DARF ICH NICHT AN DER STUDIE TEILNEHMEN?

Eine Teilnahme an der Studie ist prinzipiell nur möglich, wenn Sie **nicht gleichzeitig an einer anderen Studie teilnehmen** oder innerhalb der letzten 30 Tage teilgenommen haben.

Schwangerschaft:

Schwangere oder **stillende Patientinnen** dürfen nicht an der Studie teilnehmen. Frauen dürfen nur dann an der Studie teilnehmen, wenn das Risiko einer Schwangerschaft so gering wie möglich ist. Das heißt:

- wenn Sie chirurgisch sterilisiert sind / Ihnen die Gebärmutter entfernt wurde oder
- wenn Sie während der gesamten Studie eine wirksame und sichere Verhütungsmethode verwenden und einen negativen Schwangerschaftstest vor Beginn der Behandlung hatten.

Akzeptable Verhütungsmethoden sind:

- Spirale in Kombination mit einer *Barrieremethode* (z.B. Kondom, Frauenkondom) oder
- Hormonelle empfängnisverhütende Mittel (Pille) in Kombination mit einer *Barrieremethode* (z.B. Kondom).

Frauen im gebärfähigen Alter werden außerdem darauf hingewiesen, dass sie ab der schriftlichen Einwilligungerklärung während der gesamten Studie und bis zwei Jahre nach Abschluss der Studientherapie nicht schwanger werden dürfen. **Sollte trotz Empfängnisverhütung eine Schwangerschaft eintreten, muss dies den behandelnden Ärzten unverzüglich mitgeteilt werden.**

Männer sollten in dieser Zeit keine Kinder zeugen. Patienten, die Partnerinnen haben, die schwanger werden könnten, müssen ihre Einwilligung geben, während der gesamten Dauer bis 2 Jahre nach Beendigung der klinischen Prüfung für eine Empfängnisverhütung (z.B. Kondome) zu sorgen.

11 AUSSCHEIDEN AUS DER STUDIE

Die Teilnahme an der klinischen Prüfung ist freiwillig und setzt Ihre schriftliche Einwilligung voraus. Sie können die Teilnahme jederzeit ohne Angabe von Gründen abbrechen. Dadurch entstehen Ihnen in der weiteren Behandlung keine Nachteile. Auch das Vertrauensverhältnis zwischen Ihnen und Ihrem Arzt nimmt keinen Schaden.

Bitte informieren Sie Ihren Prüfarzt, wenn Sie nicht mehr an der Studie teilnehmen möchten. Sollte dies der Fall sein, bitten wir Sie in Ihrem eigenen Interesse, eine Abschlussuntersuchung durchführen zu lassen. Der Prüfarzt wird dann mit Ihnen besprechen, wie und wo Ihre weitere Behandlung stattfindet.

Unter gewissen Umständen ist es aber auch möglich, dass der Prüfarzt oder der Auftraggeber der Studie entscheiden, Ihre Teilnahme an der klinischen Prüfung oder die Behandlung mit den Studienmedikamenten vorzeitig zu beenden, ohne dass Sie auf die Entscheidung Einfluss haben. Die Gründe hierfür können z. B. sein, dass Ihre weitere Teilnahme an der klinischen Prüfung ärztlich nicht mehr vertretbar ist. Es kann auch passieren, dass die gesamte klinische Prüfung abgebrochen wird.

12 VERSICHERUNGSSCHUTZ

Bei der klinischen Prüfung eines Arzneimittels sind alle Studienteilnehmer gemäß dem Arzneimittelgesetz versichert. Der Versicherungsschutz erstreckt sich auf alle Gesundheitsschädigungen, die als Folge studienspezifischer Maßnahmen im Zeitraum von bis zu fünf Jahren nach Abschluss Ihrer Teilnahme eintreten. Die Maßnahmen, die zur Routinebehandlung Ihrer Erkrankung gehören, sowie die erwarteten und möglichen Nebenwirkungen der Therapie fallen nicht unter den Versicherungsschutz.

Alle Teilnehmer an der klinischen Prüfung sind bei folgender Gesellschaft versichert:

Kontaktdaten der Probanden-Versicherung:

Zurich Insurance plc
60486 Frankfurt am Main
Telefon: 069-7115-0
Fax: 069-7115-3358

Ecclesia Vers.dienst GmbH
Klingenbergtrasse 4
32758 Detmold
Tel. 05231 – 6030
Fax 05231 - 603197

Versicherungsnummer: 800.520.020.990

Um Ihren Versicherungsschutz nicht zu gefährden, beachten Sie folgende Punkte:

- Halten Sie sich genau an die Anweisungen Ihres Prüfarztes und die des Studienpersonals.
- Während der Dauer der klinischen Prüfung dürfen Sie sich einer anderen medizinischen Behandlung nur nach Rücksprache mit dem Prüfarzt unterziehen. Dies gilt selbstverständlich nicht für Notfälle. Allerdings muss Ihr Prüfarzt in diesem Fall unverzüglich von der anderen Behandlung unterrichtet werden.
- Zeigen Sie jede Gesundheitsschädigung, die als Folge der klinischen Prüfung eingetreten sein könnte, sofort dem Versicherer an, gegebenenfalls mit Unterstützung durch Ihren Prüfarzt.
- Im Schadensfall haben Sie alle zweckmäßigen Maßnahmen zu treffen, die der Aufklärung der Ursache und des Umfangs des eingetretenen Schadens sowie dessen Minderung dienen.

- Im Schadensfall sind alle behandelnden Ärzte zu ermächtigen, der Versicherungsgesellschaft auf deren Verlangen Auskunft zu erteilen.
- 30 Tage vor, während und 30 Tage nach der Durchführung der Studie dürfen Sie an keiner anderen Studie teilnehmen, da sonst der Versicherungsschutz gefährdet ist.

Die detaillierten Versicherungsbedingungen mit weiteren Einzelheiten zum Versicherungsschutz und allem, was in diesem Zusammenhang wichtig für Sie ist, wird Ihnen der Prüfarzt aushändigen, wenn Sie sich für eine Teilnahme an der Studie entscheiden.

13 WO FINDE ICH WEITERE INFORMATIONEN ZU DIESER STUDIE?

Diese Studie ist im Deutschen Register Klinischer Studien (DRKS) registriert. Über die Internetseite http://www.drks.de, Studiennummer DRKS00005503 können Informationen über diese Studie eingesehen werden. Auf der genannten Internetseite sind keine persönlichen Daten hinterlegt, mit denen Sie identifiziert werden könnten. Allenfalls werden die Ergebnisse dieser Studie dort veröffentlicht. Sie können die Internetseite jederzeit aufrufen.

14 DATENSCHUTZ

Die im Rahmen der Studie erhobenen Daten werden mit Nennung Ihres vollständigen Geburtsdatums, ohne Nennung des Namens oder Ihrer *Initialen*, gesammelt, gespeichert und ausgewertet. Die Datenübertragung erfolgt verschlüsselt und *pseudonymisiert*. Personenbezogene Daten werden gemäß den nationalen gesetzlichen Bestimmungen archiviert. Wir versichern Ihnen, dass keinerlei personenbezogene Daten im Rahmen einer wissenschaftlichen Publikation der Studie veröffentlicht werden. Die Bestimmungen der ärztlichen Schweigepflicht und des Datenschutzes sind gewährleistet. Sie haben das Recht, Ihre persönlichen Daten einzusehen und unzutreffende Angaben zu korrigieren.

Um eine zentrale radiologische Referenzbegutachtung zu erhalten, ist es nötig, dass wir CDs von den durchgeführten Kernspintomographien an unser Referenzzentrum Kantonsspital St. Gallen versenden. Die CDs sind *pseudonymisiert* verschlüsselt, das heißt, sie enthalten lediglich Ihre Studiennummer und Ihr Geburtsdatum.

Das Arzneimittelgesetz enthält nähere Vorgaben für den erforderlichen Umfang der Einwilligung in die Datenerhebung und -verwendung. Einzelheiten entnehmen Sie bitte der Einwilligungserklärung, die im Anschluss an diese Patienteninformation abgedruckt ist. Sollten Sie unter den dort genannten Bedingungen nicht einwilligen, können Sie an der klinischen Prüfung nicht teilnehmen.

15 PROBENAUFBEWAHRUNG

Blut-/Gewebeproben/Aufnahmen von bildgebenden Verfahren

Wir möchten Sie hiermit über eine **wissenschaftliche Begleitstudie** informieren.

Dabei handelt es sich um die Aufbereitung und Lagerung von Blut- und Gewebeproben, die Ihnen im Rahmen der Studienbehandlung in der Routinediagnostik sowie teilweise auch zusätzlich abgenommen werden. Sie werden über die Details gesondert informiert und haben die Möglichkeit, separat über eine Teilnahme zu entscheiden. Sollten Sie nicht in die Bereitstellung Ihrer Proben einwilligen, wird dies keinerlei Einfluss auf Ihre Teilnahme an der hier beschriebenen klinischen Prüfung haben.

Die im Rahmen der Hauptstudie entnommenen Gewebeproben sollen nach Abschluss der Untersuchungen im Rahmen der Begleitstudie anschließend eingelagert und für die Erforschung Ihrer Erkrankung weiter verwendet werden.

16 KONTAKT BEI WEITEREN FRAGEN / EIGENE NOTIZEN

Wenn Sie noch weitere Fragen haben, so wenden Sie sich bitte an Ihren Studienarzt.

**Leiter der klinischen Prüfung**	**Prüfarzt vor Ort** (bitte hier Kontaktdaten Zentrum eintragen):	
**Prof. Dr. med. Gerald Illerhaus,**<br>**Ärztlicher Direktor**<br>Klinik für Hämatologie, Onkologie<br>Stuttgart Cancer Center,<br>Tumorzentrum Eva Mayr-Stihl<br>Klinikum Stuttgart<br>Kriegsbergstraße 60<br>70174 Stuttgart	Name	
	Klinik	
	Abteilung	
	Station	
	Straße	
	PLZ Ort	
	Telefon	
	Name und<br>Tel. des Vertreters	

Des Weiteren hat die zuständige Bundesoberbehörde eine Kontaktstelle für alle Studienteilnehmer und deren gesetzlichen Vertreter oder Bevollmächtigten unter folgender Adresse eingerichtet:

Bundesinstitut für Arzneimittel und Medizinprodukte/Fachgebiet Klinische Prüfung/Inspektionen - BfArM

Kurt-Georg-Kiesinger-Allee 3

53175 Bonn

Telefon: 0228 / 207- 4318 Fax: 0228 / 207- 4355

E-mail: klinpruefung@bfarm.de

NOTIZEN

Hier haben Sie Platz, sich Fragen zu notieren, die Sie gerne mit Ihrem behandelnden Arzt besprechen möchten.

17 GLOSSAR

**Autologe Stammzelltransplantation**	Körpereigene, zuvor gesammelte Stammzellen, werden dem Körper über einen zentralvenösen Zugang zurückgegeben.
**Barrieremethoden**	Verhütungsmethoden, die den Samenzellen wie Barrieren und Hindernisse den Weg zur Eizelle versperren (z.B. Kondom, Diaphragma).
**Chemotherapie / Chemotherapeutika**	Medikamentöse Therapie mit Stoffen, die ihre schädigende Wirkung möglichst gezielt auf bestimmte krankheits-verursachende Zellen ausüben und diese abtöten oder in ihrem Wachstum hemmen.
**Computertomographie**	Röntgenverfahren, bei dem der menschliche Körper in Querschnittbildern dargestellt wird.
**R-DeVIC -> Rituximab - Dexamethason- VP-16 (Etoposid) - Iphosphamid - Carboplatin**	Kombination von verschiedenen Medikamenten, die für die Behandlung von ZNS-Lymphomen eingesetzt werden.
**Ethik-Komission**	Gremium, das ethische und rechtliche Zulässigkeiten im Zusammenhang mit medizinischer Forschung an Studienteilnehmern klärt.
**G-CSF Neupogen**	Wachstumsfaktor, der im Knochenmark die Reifung von weißen Blutkörperchen fördert, die wichtige Funktionen im Immunsystem wahrnehmen.
**Hochdosis-Chemotherapie**	Besonders hoch dosierte Chemotherapie (siehe dort), mit der alle Krebszellen zerstört werden sollen.
**Immuntherapie**	Therapie, über die das körpereigene Abwehrsystem beeinflusst wird.
**Induktions-Chemotherapie**	Einleitende Behandlung, die die Tumorzellen akut zurück-drängt.
**Injektion**	Einbringen einer Substanz in einen Organismus über eine Kanüle mit Hilfe einer Spritze.
**Initialen**	Anfangsbuchstaben von Vor- und Nachname
**Intravenöse Infusion**	Das Medikament wird direkt über eine Vene dem Körper zugeführt.
**Kernspintomographie**	Ein bildgebendes Verfahren, welches mit Hilfe von Magnetfeldern und Radiowellen zur Darstellung von Geweben und Organen eingesetzt wird; häufig auch Magnetresonanztherapie (MRT) genannt (siehe dort).
**Konsolidierung**	Gabe von Arzneimitteln zur Aufrechterhaltung des durch die einleitende Behandlung (Induktionstherapie) herbeigeführten Ergebnisses.
**Kontrastmittel**	Einsatz zur Verbesserung der Darstellung von Funktionen und Strukturen des Körpers bei bildgebenden Verfahren wie beispielsweise Kernspintomographie und Röntgenuntersuchungen.
**Leukapherese**	Stammzellgewinnung
**Leukovorin**	Medikament, das eingesetzt wird, um die Nebenwirkungen der Chemotherapie mit dem Medikament Methotrexat auf das Knochenmark und die Schleimhäute zu verringern.
**Lymphozyten**	Zelluläre Bestandteile des Blutes, die einen Teil der sogenannten "weißen Blutkörperchen" ausmachen.
**Malignes Non-Hodgkin Lymphom**	Bösartige Erkrankung des lymphatischen Systems
**MATRix -> Methotrexat (MTX) - Cytarabin (Ara-C) - Thiotepa - Rituximab**	Medikamente, die breiten Einsatz in der Behandlung von bösartigen Erkrankungen des Blutes und des lymphatischen Systems finden und bei primär zerebralem Lymphom als besonders wirksam gelten.

**MRT**	Magnetresonanztomographie, siehe auch Kernspin-tomographie.
**Multizentrische Phase III Studie**	Studie, die in mehreren Kliniken durchgeführt wird mit zugelassenen Medikamenten, die in einer neuen Kombination mit anderen zugelassenen Medikamenten gegeben werden zur Therapieoptimierung.
**Pseudonymisiert**	Der Patientenname wird durch einen Zahlencode ersetzt, sodass die Identifikation des Betroffenen erschwert, bzw. für Außenstehende nicht nachvollziehbar ist.
**Randomisierung**	Verfahren, bei dem der teilnehmende Patient unter Verwendung eines Zufallsmechanismus unterschiedlichen Behandlungsgruppen zugeordnet wird.
**Registrierung**	Zentrale Erfassung des Patienten als Studienteilnehmer.
**Rituximab**	Eine Form der Immuntherapie (siehe oben unter Immuntherapie), die sich gegen die bösartigen Lymphomzellen richtet.
**Stammzelle**	Eine Stammzelle ist eine Art Ursprungszelle, die sich unbegrenzt vermehren und alle Zelltypen des Körpers bilden kann (zum Beispiel Muskelzelle, Nervenzelle, Blutzelle).
**Thiotepa**	Medikament, das Einsatz in der Behandlung von bösartigen Erkrankungen des lymphatischen Systems findet und bei primär zerebralem Lymphom als sehr wirksam gilt.
**Transfusion**	Übertragung von Blut oder Blutbestandteilen, die aus dem Blut eines menschlichen Blutspenders gewonnen werden.
**Zentralvenöser Katheter**	Bei der Legung eines zentralvenösen Zugangs wird ein dünner Kunststoffschlauch (Katheter) in eine große Körpervene, meist im Hals oder im oberen Brustkorb, gelegt. Durch diesen Zugang können dem Patienten dann Medikamente oder Nährstoffe zugeführt oder Blut abgenommen werden. Häufige Venenpunktionen können so vermieden werden.
**Zyklus**	Behandlungsabschnitt
**Zytostatika**	Natürliche oder synthetische Substanzen, die das Zellwachstum beziehungsweise die Zellteilung hemmen. Sie werden vor allem zur Behandlung von bösartigen Tumoren eingesetzt.

Platzhalter Patientenetikett

EINWILLIGUNGSERKLÄRUNG

Studientitel:

Randomisierte, kontrollierte, offene, multizentrische Phase III Studie mit 2 parallelen Armen - Hochdosis Chemotherapie und autologe Stammzelltransplantation oder konventionale Chemotherapie zur Konsolidierung bei primären ZNS Lymphomen – (Methotrexat, Ara-C, Thiotepa, Rituximab)

Studienkurztitel: MATRix / IELSG43

EudraCT-Nr. 2012-000620-17

Zentrums- und Patienten-Nr.	Patienten-Name	Geburtsdatum
__ __ / __ __ __		

Ich wurde vom Prüfer

Frau / Herrn:	Tel.:

mündlich und schriftlich über die Ziele, den Ablauf der Studie sowie über die zu erwartenden Wirkungen, über mögliche Vor- und Nachteile, über eventuelle Risiken sowie über meine Rechte informiert.

Ich habe die schriftliche Information für Patienten gelesen und verstanden. Meine Fragen im Zusammenhang mit der Teilnahme an dieser Studie sind mir zufriedenstellend beantwortet worden. Zusätzlich wurden folgende Punkte besprochen:

....

....

Ich bin darüber informiert, dass eine studienspezifische Versicherung alle Gesundheitsschäden deckt, die im Rahmen der Studie auftreten könnten. Ich weiß, dass ich während der Dauer der Studie vor Beginn einer anderen medizinischen Behandlung – außer in Notfällen – Rücksprache mit meinem Studienarzt treffen sollte. Eine Gesundheitsschädigung, die als Folge der Studie eingetreten sein könnte, muss ich dem Versicherer unverzüglich anzeigen (Gewährleistung des Versicherungsschutzes). Zum diesem Zweck kann ich mich auch an meinen Arzt wenden.

Ich erkläre mich damit einverstanden, dass ich alle Hinweise zum Ablauf dieser Studie befolgen werde und ich werde den Prüfarzt über jede auftretende Erkrankung oder Beschwerde informieren.

Ich erkläre hiermit meine Einwilligung, an dieser klinischen Studie freiwillig teilzunehmen. Mir ist bekannt, dass ich meine Einwilligung jederzeit ohne Angabe von Gründen widerrufen kann, ohne dass mir nachteilige Folgen für meine weitere medizinische Versorgung entstehen.

Sollten die Daten für wissenschaftliche Zwecke oder für eine Publikation genutzt werden, so ist kein Rückschluss auf meine Person möglich.

ERKLÄRUNG ZUM DATENSCHUTZ

Mir ist bekannt, dass bei dieser klinischen Prüfung personenbezogene Daten, insbesondere medizinische Befunde über mich erhoben, gespeichert und ausgewertet werden sollen. Die Verwendung der Angaben über meine Gesundheit erfolgt nach gesetzlichen Bestimmungen und setzt vor der Teilnahme an der klinischen Prüfung folgende freiwillig abgegebene Einwilligungserklärung voraus, das heißt ohne die nachfolgende Einwilligung kann ich nicht an der klinischen Prüfung teilnehmen.

1. Ich erkläre mich damit einverstanden, dass im Rahmen dieser klinischen Prüfung personenbezogene Daten, insbesondere Angaben über meine Gesundheit auf Fragebögen, Dokumentationsbögen und elektronischen Datenträgern aufgezeichnet und nach einer Verschlüsselung (*Pseudonymisierung*), die keine Rückschlüsse auf meine Identität zulassen, weitergegeben werden:

   a. für die Zwecke einer wissenschaftlichen Analyse an den Auftraggeber, das Klinikum Stuttgart, Prof. Dr. med. G. Illerhaus, Ärztlicher Direktor Klinik f. Hämatologie und Onkologie bzw. dessen Vertragsnehmer, das Universitätsklinikum Freiburg. Abteilung Medizin I und das Studienzentrum,

   b. an die jeweils zuständige Ethikkommission und Bundesoberbehörde (Bundesinstitut für Arzneimittel und Medizinprodukte), und ggf. an ausländische Behörden zur ordnungsgemäßen Durchführung der Studie, zur Überwachung der Arzneimittelsicherheit und der Studienergebnisse im Rahmen der gesetzlichen Verpflichtungen sowie zur Überprüfung der Zulassungsunterlagen. Sollten schwerwiegende unerwünschte Ereignisse auftreten, so wird mein Geburtsdatum aus Gründen der Sicherheit ebenfalls registriert.

2. Außerdem erkläre ich mich damit einverstanden, dass autorisierte und zur Verschwiegenheit verpflichtete Beauftragte von dem Klinikum Stuttgart bzw. dessen Vertragsnehmer sowie die zuständigen Überwachungsbehörden in meine beim Prüfarzt vorhandenen personenbezogenen Daten, insbesondere meine Gesundheitsdaten, Einsicht nehmen, soweit dies für die Überprüfung der ordnungsgemäßen Durchführung der Studie notwendig ist. Für diese Maßnahme entbinde ich den Prüfarzt von der ärztlichen Schweigepflicht.

3. Ich bin darüber aufgeklärt worden, dass ich jederzeit die Teilnahme an der klinischen Prüfung beenden kann. Die Einwilligung zur Erhebung und Verarbeitung meiner personenbezogenen Daten, insbesondere der Angaben über meine Gesundheit, ist jedoch unwiderruflich. Ich weiß, dass im Falle eines Widerrufs zur Teilnahme an der klinischen Prüfung die bis zu diesem Zeitpunkt gespeicherten Daten weiterhin verwendet werden dürfen, soweit dies erforderlich ist, um

   a) Wirkungen des zu prüfenden Arzneimittels festzustellen,

   b) sicherzustellen, dass meine schutzwürdigen Interessen nicht beeinträchtigt werden,

   c) der Pflicht zur Vorlage vollständiger Zulassungsunterlagen zu genügen.

4. Ich erkläre mich damit einverstanden, dass meine Daten nach Beendigung oder Abbruch der Prüfung entsprechend den nationalen gesetzlichen Vorschriften (oder studien-/länderspezifische Angaben z.B. 10/15 Jahre) aufbewahrt werden. Danach werden meine personenbezogenen Daten gelöscht, soweit nicht gesetzliche, satzungsmäßige oder vertragliche Aufbewahrungsfristen entgegenstehen.

5. Ich bin über folgende gesetzliche Regelung informiert: Falls ich meine Einwilligung widerrufe, an der Studie teilzunehmen, müssen alle Stellen, die meine personenbezogenen Daten, insbesondere Gesundheitsdaten, gespeichert haben, unverzüglich prüfen, inwieweit die gespeicherten Daten für die in Nr. 3 a) bis c) genannten Zwecke noch erforderlich sind. Nicht mehr benötigte Daten sind unverzüglich zu löschen.

6. Ich bin damit einverstanden, dass Gesundheitsdaten bei mitbehandelnden Ärzten erhoben oder eingesehen werden, soweit dies für die ordnungsgemäße Durchführung und Überwachung der Studie notwendig ist. Insoweit entbinde ich diese Ärzte von der Schweigepflicht. (Falls nicht gewünscht, bitte streichen.)

7. Ich bin damit einverstanden, dass mein Hausarzt über meine Teilnahme an der klinischen Prüfung informiert wird (falls dies nicht gewünscht wird, bitte streichen).

Eine Kopie dieser Einwilligungserklärung und der Patienteninformation sowie die Versicherungsbedingungen habe ich erhalten.

Datum des Patienten [1)]

Unterschrift des Patienten

Datum des Prüfarztes [1)]

Unterschrift des Prüfarztes

1) Das Datum der Unterschrift ist von allen Personen eigenhändig einzutragen.

<u>Im Falle von geschäftsunfähigen Patienten:</u>

Einwilligungserklärung durch einen gesetzlichen Vertreter oder Bevollmächtigten

Hiermit erkläre ich, dass ich als gesetzlicher Vertreter/Bevollmächtigter des o. g. Patienten in der in dieser Einwilligungserklärung angegebenen Weise und zu den in der Patienteninformation bzw. in dieser Einwilligungserklärung aufgeführten Sachverhalten schriftlich und mündlich aufgeklärt worden bin. Mir ist bekannt, dass meine Einwilligung als gesetzlicher Vertreter / Bevollmächtigter dem mutmaßlichen Willen des Patienten entsprechen muss und bestätige durch meine Unterschrift, dass dies der Fall ist.

Des Weiteren erkläre ich mit meiner Unterschrift meine Einwilligung, dass o. g. Patient an dieser Studie teilnimmt. Außerdem erkläre ich, dass für mich mit dieser Einwilligungserklärung keinerlei Vorteile verbunden sind.

Datum (eigenhändig einzutragen)

Unterschrift des gesetzlichen Vertreters / des Bevollmächtigten
(Unzutreffendes bitte streichen)

Patientenetikett	Stempel der Einrichtung

A U F K L Ä R U N G und E I N W I L L I G U N G S E R K L Ä R U N G

über Probenentnahmen zu Forschungszwecken bei Verdacht auf eine Tumor-Erkrankung (hämatologisch oder onkologisch)

Sehr geehrte Patientin, sehr geehrter Patient!

Sie befinden sich wegen einer Tumorerkrankung in medizinischer Betreuung. Die Erforschung solcher Erkrankungen bildet die Grundlage für ein genaueres Verständnis der Krankheitsprozesse sowie für die Erarbeitung neuer Behandlungsmethoden.

Ziel ist es, mit neuen diagnostischen Methoden eine bessere Klassifikation der Erkrankung und eine verbesserte Prognosestellung bezüglich des Ansprechens auf Therapien zu ermöglichen. Hierzu werden Zell- und Gewebeproben der Patienten in einer Probenbank gelagert. Neue Verfahren erlauben die gleichzeitige Charakterisierung von Tausenden von Eiweißen sowie der Erbinformation.

Die Untersuchungen können auch mit Blutproben durchgeführt werden. Wir möchten durch solche Untersuchungen feststellen, welche Veränderungen der Eiweißzusammensetzung der Zellen bei Ihrer Erkrankung vorliegt und wie sie den Krankheitsverlauf beeinflussen. Die zu den Proben gespeicherten Daten und die aus den Proben gewonnenen Ergebnisse werden so verschlüsselt, dass die Identifizierung persönlicher Kennzeichnung, die einen Rückschluss auf Ihre Person erlaubt, nicht möglich ist. Wenn sich aber aus der Analyse Ihrer Blutprobe und der systematischen Auswertung gleichartiger Proben wichtige Erkenntnisse für Ihren Krankheitsverlauf oder Ihre Behandlung ergeben, kann mit Ihrer Einwilligung ein Rückgriff auf Ihre persönlichen Daten erfolgen.

Wir möchten Sie daher um Ihre Einwilligung bitten, dass wir von den ohnehin zur Diagnostik notwendigen Probenentnahmen zusätzlich Probenmaterial lagern und es für entsprechende Untersuchungen reinigen dürfen. Sie haben das Recht, diese Lagerung abzulehnen und jederzeit die Möglichkeit, Ihre Einwilligung zurückzunehmen. In diesem Fall würden bereits aufbereitete Materialien vernichtet und alle Daten gelöscht werden.

Speziell angesprochene Punkte:

………

………

………

Einwilligungserklärung

Ich habe die ärztliche Aufklärung über die zusätzliche Probengewinnung verstanden und erkläre mich mit der Entnahme, Lagerung und Verwendung der entnommenen Proben zu Forschungszwecken einverstanden.

Kommerzielle Verwertung der Forschungsergebnisse:

Einwilligung für den seltenen Fall, dass die Forschungsergebnisse zu einer kommerziellen Verwertung durch das Klinikum führen. (bitte Zutreffendes ankreuzen)

☐ Ja, ich willige ein in die kommerzielle Verwertung der Forschungsergebnisse.

☐ Nein, ich willige nicht ein in die kommerzielle Verwertung der Forschungsergebnisse.

Ich erkläre, dass mir alle diesbezüglichen Fragen hinreichend beantwortet wurden. Ich bin darüber informiert, dass ich meine heute gegebene Einwilligung jederzeit und ohne Angabe von Gründen widerrufen kann, ohne dass mir hieraus ein Nachteil entsteht. Ich versichere, dass ich mich zum Zeitpunkt meiner Unterschrift im vollen Besitz meiner geistigen Kräfte befinde und diese Entscheidung aus meinem freien Willen treffe.

Ich bin mit der Aufzeichnung von Krankheitsdaten bezüglich meiner Grunderkrankung und Therapie in anonymisierter Form in wissenschaftlichen Veröffentlichungen einverstanden. Ich wurde darüber informiert, dass beim Umgang mit meinen Daten die Grundsätze des Datenschutzes (Anonymität, Datensicherung) beachtet werden. Mit der elektronischen Verarbeitung der bei mir erhobenen Daten bin ich einverstanden.

Ort, Datum:………………………………………………………………

………

Patient/Patientin (Druck- und Unterschrift) Arzt/Ärztin (Unterschrift u. Stempel)

<u>Im Falle von geschäftsunfähigen Patienten:</u>

Einwilligungserklärung durch einen gesetzlichen Vertreter oder Bevollmächtigten

Hiermit erkläre ich, dass ich als gesetzlicher Vertreter/Bevollmächtigter des o. g. Patienten in der in dieser Einwilligungserklärung angegebenen Weise und zu den in der Patienteninformation bzw. in dieser Einwilligungserklärung aufgeführten Sachverhalten schriftlich und mündlich aufgeklärt worden bin. Mir ist bekannt, dass meine Einwilligung als gesetzlicher Vertreter / Bevollmächtigter dem mutmaßlichen Willen des Patienten entsprechen muss und bestätige durch meine Unterschrift, dass dies der Fall ist.

Des Weiteren erkläre ich mit meiner Unterschrift meine Einwilligung, dass o. g. Patient an dieser Studie teilnimmt. Außerdem erkläre ich, dass für mich mit dieser Einwilligungserklärung keinerlei Vorteile verbunden sind.

Datum (eigenhändig einzutragen)

Unterschrift des gesetzlichen Vertreters / des Bevollmächtigten
(Unzutreffendes bitte streichen)

Biological specimens	33	Processing the primary tumor samples available in the study biobank we are planning to analyse the genomic profile, mRNA and miRNA expression. A multivariate analysis investigating the correlation of the molecular findings with data regarding the response to therapy, the clinical course and the overall survival of the patients should be conducted.<br>Processing the blood and bloodserum samples available in the study biobank, established prognostic factors (e.g. lactate dehydrogenase levels) should be verified and unknown factors should be identified.<br>Processing the CSF samples available in the study biobank the observation that certain miRNAs can be used for diagnosis and molecular monitoring of PCNSL should be verified in a prospective manner.

Trial Flow Chart 34

Flow Chart

Visit schedule and assessments[1]	Screen period<br>d -16 until d -2	Regis-tration<br>d -1	Induction treatment: four 3-week cycles[1] (d0 to d20)						Re-Check[2]/ Random ization	Consolidation treatment[1]			EOT Visit	Follow up	
										Arm A: R-DeVIC two 3-week cycles		Arm B: HDT-ASCT (day -8 to day 0)			
			Visit 1	Visit 2	RA I	Visit 3	Visit 4	RA II		Visit 5	Visit 6	Visit 5	RA III	Yr 1-2	Yr *** 3-5
			day 0 to day 5 of cycle 1**	day 0 to day 5 of cycle 2**	d 18 to day 20 of cycle 2	day 0 to day 5 of cycle 3**	day 0 to day 5 of cycle 4**	d 18 to day 20 of cycle 4		day 0 of cycle 1	day 0 of cycle 2	Start of HDT	day 60 after random ization	every 3mo	every 6mo
Informed consent/ Demographic data[3]	X														
Inclusion/ Exclusion criteria	X								X						
Registration/ Randomization		X							X						
Medical history, height	X														
Pregnancy test (serum beta-hCG)	X							X					X	X****	
Treatment administration			X	X		X	X			X	X	X			
ECOG	X		X	X		X	X			X	X	X	X	X	X
MMSE, QOL (EORTC QLQ-C30, -BN20)	X							X					X	X[5]	X[5]
Neuropsychological battery[4]	X												X	X[5]	X[5]
Weight	X		X	X		X	X			X	X	X			
Vital signs*, physical and neurological examination	X		X*	X*		X*	X*			X*	X*	X*	X*	X*	X*
Hematology[6]* / clinical chemistry[7]*	X		X	X		X	X			X	X	X	X	X	X
Creatinine, estimated GFR (MDRD)			X	X		X	X			X	X	X			
LDH	X														
Hepatitis B/C serology, HIV test*	X														
Whole body plethysmography*	X											X	X[8]		
Electrocardiography*	X									X		X	X		
Echocardiography*	X														
Testicular ultrasound*	X														
Abdominal ultrasound*			X	X		X	X								
Whole body CT scan[9]*	X														
Whole brain MRI	X				X			X					X	X	X
Central pathology	X								X[14]						
BM examination *	X														
Slit lamp examination	X				X[11]			X[11]					X[11]		
CSF examination[10]	X				X[11]			X[11]					X[11]		
Translational program	X[12]												X[13]		
Concomitant medication	X (see section 6.6)														
Adverse events/toxicity CTCAE			X (see section 10.1 and 10.1.2)												

Participating centers	35

MATRix / IELSG43 Studie:
EudraCT-Nummer: 2012-000620-17

Participating centers und ethics committees

	Leading center	Principal Trial Coordinator	Leading ethics committee
1	**Klinikum Stuttgart<br>Klinik für Hämatologie, Onkologie und Palliativmedizin<br>Stuttgart Cancer Center / Tumorzentrum Eva Mayr-Stihl<br>Kriegsbergstr. 60<br>70174 Stuttgart**	**LKP:<br>Prof. Dr. Gerald Illerhaus<br>Stellvertreterin:<br>Dr. Kristina Mikesch**	**Ethik-Kommission bei der Landesärztekammer Baden-Württemberg<br>Jahnstraße 40<br>70597 Stuttgart**
	**Participating centers**	**PI & Deputy PI:**	**Local ethics committees:**
2	Universitätsklinikum Aachen<br>Klinik für Onkologie, Hämatologie und Stammzelltransplantation<br>(Medizinische Klinik IV)<br>Pauwelsstr. 30<br>52074 Aachen	Prüfer:<br>Dr. med. Jens Panse<br>Stellvertreterin:<br>Dr. med. Mareike Tometten	Ethik-Kommission an der Medizinischen Fakultät<br>der RWTH Aachen<br>Pauwelsstraße 30<br>52074 Aachen
3	Klinikum Augsburg<br>II. Medizinische Klinik<br>Cancer Center Augsburg<br>Stenglinstr.2<br>86156 Augsburg	Prüfer:<br>Prof. Dr. Martin Trepel<br>Stellvertreter:<br>PD Dr. Andreas Rank	Ethik-Kommission der Bayerischen Landesärztekammer<br>Mühlbaurstraße 16<br>81677 München
4	Städtisches Klinikum Braunschweig gGmbH<br>Medizinische Klinik III<br>Hämatologie und Onkologie<br>Celler Straße 38<br>38114 Braunschweig	Prüfer:<br>Dr. Sebastian Schroll<br>Stellvertreter:<br>Dr. Heiko Hütten	Ethik-Kommission bei der Ärztekammer Niedersachsen<br>Berliner Allee 20<br>30175 Hannover
5	Klinikum Bremen-Mitte gGmbH<br>Medizinische Klinik I<br>St.Jürgen.Str. 1<br>28177 Bremen	Prüfer:<br>Prof. Dr. Bernd Hertenstein<br>Stellvertreter:<br>Dr. Stefan Kaun	Ethik-Kommission des Landes Bremen<br>Institut für klinische Pharmakologie<br>Klinikum Bremen-Mitte gGmbH<br>St.-Jürgen-Straße 1<br>28177 Bremen
6	Klinikum Chemnitz gGmbH<br>Klinik für Innere Medizin III<br>Bürgerstraße 2<br>09113 Chemnitz	Prüfer:<br>PD Dr. Mathias Hänel<br>Stellvertreterin:<br>Dr. Anke Morgner	Ethik-Kommission der sächsischen Landesärztekammer<br>Schützenhöhe 16<br>01099 Dresden
7	Universitätsklinikum Düsseldorf<br>Klinik für Hämatologie, Onkologie und Klinische Immunologie<br>Moorenstr. 5<br>40225 Düsseldorf	Prüfer:<br>Prof. Dr. Guido Kobbe<br>Stellvertreter:<br>Dr. Mustafa Kondakci	Ethik-Kommission der Medizinischen Fakultät der Heinrich-Heine-Universität Düsseldorf<br>Kinderklinik, Geb. 13.41<br>Moorenstr. 5<br>40225 Düsseldorf
8	Universitätsklinikum Erlangen<br>Medizinische Klinik 5<br>Hämatologie und Internistische Onkologie<br>Ulmenweg 18<br>91054 Erlangen	Prüfer:<br>Prof. Dr. Stephan Krause<br>Stellvertreter:<br>Dr. Bernd Spriewald	Ethik-Kommission der Medizinischen Fakultät<br>Friedrich-Alexander-Universität Erlangen-Nürnberg<br>Krankenhausstraße 12<br>91054 Erlangen
9	Universitätsklinikum Essen<br>Klinik für Hämatologie<br>Hufelandstraße 55<br>45122 Essen	Prüfer:<br>PD Dr. Alexander Röth<br>Stellvertreter:<br>PD Dr. Andreas Hüttmann	Ethik-Kommission der Med. Fakultät der Universität Duisburg-Essen<br>Jakobs Universitätsklinikum Essen<br>Robert-Koch-Str. 9-11<br>45122 Essen

MATRix / IELSG43 Studie:
EudraCT-Nummer: 2012-000620-17

Participating centers und ethics committees

10	Klinikum der Johann-Wolfgang-Goethe-Universität<br>Medizinische Klinik II<br>Hämatologie und Onkologie<br>Theodor-Stern-Kai 7<br>60590 Frankfurt/Main	Prüfer:<br>Dr. Johannes Atta<br>Stellvertreterin:<br>Dr. Uta Brunnberg	Ethik-Kommission des Fachbereichs Medizin<br>der Johann Wolfgang Goethe-Universität Frankfurt<br>Theodor-Stern-Kai 7<br>60590 Frankfurt am Main
11	Universitätsklinikum Freiburg<br>Medizinische Klinik I<br>Hugstetter Str. 55<br>79106 Freiburg	Prüfer:<br>Prof. Dr. Jürgen Finke<br>Stellvertreterin:<br>Dr. Elisabeth Schorb	Ethik-Kommission<br>der Albert-Ludwigs-Universität Freiburg<br>Engelberger Straße 21<br>79106 Freiburg
12	Universitätsklinikum Göttingen<br>Abteilung Hämatologie/Onkologie, Neurochirurgie<br>Robert-Koch-Str. 40<br>37075 Göttingen	Prüfer:<br>Dr. med. Justin Hasenkamp<br>Stellvertreterin:<br>Dr. med. Friederike Braulke	Ethik-Kommission der Medizinischen Fakultät der<br>Georg-August-Universität<br>Von-Siebold-Straße 3<br>37075 Göttingen
13	Universitätsmedizin Greifswald<br>Klinik und Poliklinik für Innere Medizin C<br>Hämatologie, Onkologie und Transplantationszentrum<br>Sauerbruchstraße<br>17475 Greifswald	Prüfer:<br>Prof. Dr. Christian Andreas Schmidt<br>Stellvertreter:<br>Dr. Carsten Hirt	Ethikkommission an der<br>Universitätsmedizin Greifswald<br>Institut für Pharmakologie<br>Felix-Hausdorff-Str.3<br>17487 Greifswald
14	Universitätsklinikum Halle (Saale)<br>Klinik für Innere Medizin IV<br>Hämatologie/ Onkologie<br>Ernst-Grube-Straße 40<br>06120 Halle	Prüferin:<br>PD Dr. Katrin Jordan<br>Stellvertreter:<br>PD Dr. Lutz Müller	Ethik-Kommissionen der Medizinischen Fakultät<br>der Martin-Luther-Universität Halle-Wittenberg<br>Magdeburger Str. 16<br>06112 Halle (Saale)
15	Asklepios Klinik Altona<br>Hämatologie, internistische Onkologie und Palliativmedizin<br>Paul-Ehrlich-Straße 1<br>22763 Hamburg	Prüfer:<br>Prof. Dr. Christian Meyer zum Büschenfelde<br>Stellvertreter:<br>Dr. Murwan Ayoub	Ethik-Kommission der Ärztekammer Hamburg<br>Weidestraße 122 b<br>22083 Hamburg
16	Universitätskrankenhaus Hamburg-Eppendorf<br>Medizinische Klinik II<br>Onkologisches Zentrum<br>Martinistr. 52<br>20246 Hamburg	Prüferin:<br>PD Dr. Mascha Binder<br>Stellvertreter:<br>Dr. Winfried Alsdorf	Ethik-Kommission der Ärztekammer Hamburg<br>Weidestraße 122 b<br>22083 Hamburg
17	Medizinische Hochschule Hannover<br>Klinik für Hämatologie, Hämostaseologie, Onkologie und Stammzelltransplantation<br>Carl-Neuberg-Str. 1<br>30625 Hannover	Prüferin:<br>Prof. Dr. Anke Franzke<br>Stellvertreter:<br>PD Dr. Christian Könecke<br>Stellvertreterin:<br>PD Dr. Felicitas Thol	Ethik-Kommission der Medizinischen Hochschule Hannover<br>Carl-Neuberg-Straße 1<br>30625 Hannover
18	Universitätsklinikum des Saarlandes Homburg<br>Innere Medizin I<br>Kirrberger Straße<br>66424 Homburg/ Saar	Prüfer:<br>Prof. Dr. Michael Pfreundschuh<br>Stellvertreter:<br>Dr. Niels Murawski	Ethik-Kommission bei der Ärztekammer des Saarlandes<br>Faktoreistr. 4<br>66111 Saarbrücken

Participating centers und ethics committees

19	Medizinische Universitätsklinik Heidelberg<br>Abteilung Innere Medizin V<br>Im Neuenheimer Feld 410<br>69120 Heidelberg	Prüferin:<br>Prof. Dr. Gerlinde Egerer<br>Stellvertreter:<br>PD Dr. Mathias Witzens-Harig	Ethik-Kommission I<br>der Medizinischen Fakultät Heidelberg<br>Alte Glockengießerei 11/1<br>69115 Heidelberg
20	Universitätsklinikum Jena<br>Klinik und Poliklinik für Innere Medizin II<br>Hämatologie und internistische Onkologie<br>Erlanger Allee 101<br>07747 Jena	Prüfer:<br>PD Dr. Thomas Ernst<br>Stellvertreter:<br>PD Dr. Sebastian Scholl	Ethik-Kommission der Friedrich-Schiller-Universität Jena an der Medizinischen Fakultät<br>Bachstraße 18<br>07740 Jena
21	Universitätsklinikum Schleswig-Holstein, Campus Kiel<br>Klinik für Innere Medizin II<br>Hämatologie und Internistische Onkologie<br>Arnold-Heller-Str. 3 (Haus 50)<br>24105 Kiel	Prüfer:<br>Prof. Dr. Dr. Michael Kneba<br>Stellvertreterin:<br>PD Dr. Christiane Pott	Ethik-Kommission der Medizinischen Fakultät der Christian-Albrechts-Universität zu Kiel<br>Arnold-Heller-Straße 3<br>Haus 9<br>24105 Kiel
22	Universitätsklinikum Schleswig-Holstein<br>II. Medizinische Klinik und Poliklinik<br>Chemnitzstr. 33<br>24116 Kiel	Prüfer:<br>Prof. Dr. Dr. Michael Kneba<br>Stellvertreterin:<br>Dr. Monika Lamprecht	Ethik-Kommission der Medizinischen Fakultät der Christian-Albrechts-Universität zu Kiel<br>Arnold-Heller-Straße 3<br>Haus 9<br>24105 Kiel
23	Stiftungsklinikum Mittelrhein GmbH<br>Zentrum für Innere Medizin<br>Johannes-Müller-Str. 7<br>56068 Koblenz	Prüfer:<br>Prof. Dr. Ralph Naumann<br>Stellvertreter:<br>Dr. Dirk Niemann	Ethik-Kommission bei der Landesärztekammer Rheinland-Pfalz<br>Deutschhausplatz 3<br>55116 Mainz
24	Universitätsklinikum Köln<br>Innere Medizin 1<br>Kerpenerstr. 62<br>50937 Köln	Prüfer:<br>Prof. Dr. Peter Borchmann<br>Stellvertreter:<br>Prof. Dr. Kai Hübel<br>Stellvertreterin:<br>Dr. Stefanie Sasse	Ethik-Kommission der Medizinischen Fakultät der Universität zu Köln<br>Gebäude 5, Kerpener Str.62<br>50937 Köln
25	Universitätsklinikum Magdeburg AöR<br>Klinik für Hämatologie und Onkologie<br>Leipziger Str. 44<br>39120 Magdeburg	Prüfer:<br>Dr. Thomas Heinicke<br>Stellvertreter:<br>Dr. Enrico Schalk	Ethik-Kommission der Otto-von-Guericke-Universität<br>an der Medizinischen Fakultät<br>Leipziger Straße 44<br>39120 Magdeburg
26	Universitätsmedizin der Johannes Gutenberg-Universität<br>III. Med. Klinik und Poliklinik<br>Langenbeckstr. 1<br>55101 Mainz	Prüfer:<br>PD Dr. Georg Heß<br>Stellvertreter:<br>Dr. Markus Munder	Ethik-Kommission bei der Landesärztekammer Rheinland-Pfalz<br>Deutschhausplatz 3<br>55116 Mainz
27	Klinikum der Universität München - Großhadern<br>Medizinische Klinik und Poliklinik III<br>Hämatologie/Onkologie<br>Marchioninistr. 15<br>81377 München	Prüfer:<br>Prof. Dr. Martin Dreyling<br>Stellvertreterin:<br>Dr. Louisa von Baumgarten	Ethik-Kommission der Medizinischen Fakultät<br>der Ludwig-Maximilians Universität, München<br>Pettenkoferstr. 8a<br>80336 München

Participating centers und ethics committees

28	Klinikum rechts der Isar<br>der TU München<br>III. Med. Klinik und Poliklinik<br>Hämatologie u. Internistische Onkologie<br>Ismaninger Straße 22<br>81675 München	Prüfer:<br>Prof. Dr. Ulrich Keller<br>Stellvertreter:<br>Dr. Alexander Esmaty	Ethik-Kommission der Fakultät<br>für Medizin der Technischen Universität München<br>Ismaninger Str. 22<br>81675 München
29	Ethik-Kommission der<br>Ärztekammer Westfalen-Lippe<br>und der Westfälischen Wilhelms-Universität Münster<br>Gartenstraße 210-214<br>48147 Münster	Prüfer:<br>Prof. Dr. Georg Lenz<br>Stellvertreterin:<br>Dr. Andrea Kerkhoff	Ethik-Kommission der Ärztekammer<br>Westfalen-Lippe und der Westfälischen<br>Wilhelms-Universität Münster<br>Gartenstraße 210-214<br>48147 Münster
30	Pius-Hospital<br>Abteilung für Internistische Onkologie<br>Georgstraße 12<br>26121 Oldenburg	Prüfer:<br>Prof. Dr. Frank Griesinger<br>Stellvertreterin:<br>Dr. Imme Conradi	Ethik-Kommission bei der<br>Ärztekammer Niedersachsen<br>Berliner Allee 20<br>30175 Hannover
31	Klinikum Oldenburg gGmbH<br>Abt. Onkologie/Hämatologie<br>Rahel-Straus-Str. 10<br>26133 Oldenburg	Prüfer:<br>Prof. Dr. Bernd Metzner<br>Stellvertreterin:<br>Dr. Ruth Thole	Ethik-Kommission bei der<br>Ärztekammer Niedersachsen<br>Berliner Allee 20<br>30175 Hannover
32	Universitätsklinikum Regensburg<br>Klinik & Poliklinik für Innere Medizin III<br>Hämatologie & Onkologie<br>Franz-Josef-Strauss-Allee 11<br>**93053 Regensburg**	Prüfer:<br>Prof. Dr. Tobias Pukrop<br>Stellvertreter:<br>Dr. Joachim Hahn	Ethik-Kommission an der Universität Regensburg<br>Franz-Josef-Strauß Allee 11<br>93053 Regensburg
33	Universitätsklinikum Tübingen<br>Medizinische Klinik II<br>Otfried-Müller-Str. 10<br>72076 Tübingen	Prüfer:<br>Prof. Dr. Robert Möhle<br>Stellvertreter:<br>Dr. Martin Sökler	Ethik-Kommission der Medizinischen<br>Fakultät und am Universitätsklinikum<br>Tübingen<br>Gartenstraße 47<br>72074 Tübingen
34	Universitätsklinikum Ulm<br>Klinik für Innere Medizin III<br>Hämatologie, Onkologie,<br>Rheumatologie und<br>Infektionskrankheiten<br>Albert-Einstein-Allee 23<br>89081 Ulm	Prüfer:<br>Prof. Dr. Stephan Stilgenbauer<br>Stellvertreterin:<br>Dr. Henriette Huber	Ethik-Kommission der Universität Ulm<br>Helmholtzstraße 20<br>89081 Ulm
35	Schwarzwald-Baar Klinikum<br>Villingen-Schwenningen GmbH<br>Klinikstr. 11<br>78052 Villingen-Schwenningen	Prüfer:<br>Prof. Dr. Wolfgang Brugger<br>Stellvertreterin:<br>Dr. Anja Rückert	Ethik-Kommission bei der<br>Landesärztekammer<br>Baden - Württemberg<br>Jahnstraße 40<br>70597 Stuttgart

1. Nelson DF. Radiotherapy in the treatment of primary central nervous system lymphoma (PCNSL). Journal of Neuro-Oncology. 1999;43(3):241-7.
2. Reni M, Ferreri AJ, Garancini MP, Villa E. Therapeutic management of primary central nervous system lymphoma in immunocompetent patients: results of a critical review of the literature. Annals of Oncology. 1997;8(3):227-34.
3. Abrey LE, DeAngelis LM, Yahalom J. Long-term survival in primary CNS lymphoma. Journal of Clinical Oncology. 1998;16(3):859-63.
4. Abrey LE, Yahalom J, DeAngelis LM. Treatment for primary CNS lymphoma: the next step. Journal of Clinical Oncology. 2000;18(17):3144-50.
5. DeAngelis LM, Seiferheld W, Schold SC, Fisher B, Schultz CJ. Combination chemotherapy and radiotherapy for primary central nervous system lymphoma: Radiation Therapy Oncology Group Study 93-10. J Clin Oncol. 2002;20(24):4643-8.
6. Ferreri AJ, Abrey LE, Blay JY, Borisch B, Hochman J, Neuwelt EA, et al. Summary statement on primary central nervous system lymphomas from the Eighth International Conference on Malignant Lymphoma, Lugano, Switzerland, June 12 to 15, 2002. Journal of Clinical Oncology. 2003;21(12):2407-14.
7. Ferreri AJ, Reni M, Villa E. Therapeutic management of primary central nervous system lymphoma: lessons from prospective trials. Annals of Oncology. 2000;11(8):927-37.
8. Bataille B, Delwail V, Menet E, Vandermarcq P, Ingrand P, Wager M, et al. Primary intracerebral malignant lymphoma: report of 248 cases. J Neurosurg. 2000;92(2):261-6.
9. Ferreri AJ, Reni M, Foppoli M, Martelli M, Pangalis GA, Frezzato M, et al. High-dose cytarabine plus high-dose methotrexate versus high-dose methotrexate alone in patients with primary CNS lymphoma: a randomised phase 2 trial. Lancet. 2009.
10. Pfreundschuh M, Kuhnt E, Trumper L, Osterborg A, Trneny M, Shepherd L, et al. CHOP-like chemotherapy with or without rituximab in young patients with good-prognosis diffuse large-B-cell lymphoma: 6-year results of an open-label randomised study of the MabThera International Trial (MInT) Group. Lancet Oncol. 2011;12(11):1013-22.
11. Illerhaus G, Marks R, Ihorst G, Guttenberger R, Ostertag C, Derigs G, et al. High-dose chemotherapy with autologous stem-cell transplantation and hyperfractionated radiotherapy as first-line treatment of primary CNS lymphoma. Journal of clinical oncology : official journal of the American Society of Clinical Oncology. 2006;24(24):3865-70.
12. Illerhaus G, Muller F, Feuerhake F, Schafer AO, Ostertag C, Finke J. High-dose chemotherapy and autologous stem-cell transplantation without consolidating radiotherapy as first-line

treatment for primary lymphoma of the central nervous system. Haematologica. 2008;93(1):147-8.
13. Kasenda B, Schorb E, Fritsch K, Finke J, Illerhaus G. Prognosis after high-dose chemotherapy followed by autologous stem-cell transplantation as first-line treatment in primary CNS lymphoma--a long-term follow-up study. Ann Oncol. 2012.
14. Soussain C, Hoang-Xuan K, Taillandier L, Fourme E, Choquet S, Witz F, et al. Intensive chemotherapy followed by hematopoietic stem-cell rescue for refractory and recurrent primary CNS and intraocular lymphoma: Societe Francaise de Greffe de Moelle Osseuse-Therapie Cellulaire. J Clin Oncol. 2008;26(15):2512-8.
15. Soussain C, Suzan F, Hoang-Xuan K, Cassoux N, Levy V, Azar N, et al. Results of intensive chemotherapy followed by hematopoietic stem-cell rescue in 22 patients with refractory or recurrent primary CNS lymphoma or intraocular lymphoma. Journal of Clinical Oncology. 2001;19(3):742-9.
16. Ferreri AJ, Dell'Oro S, Foppoli M, Bernardi M, Brandes AA, Tosoni A, et al. MATILDE regimen followed by radiotherapy is an active strategy against primary CNS lymphomas. Neurology. 2006;66(9):1435-8.
17. Ponzoni M, Issa S, Batchelor TT, Rubenstein JL. Beyond high-dose methotrexate and brain radiotherapy: novel targets and agents for primary CNS lymphoma. Ann Oncol. 2013.
18. Rubenstein JL, Gupta NK, Mannis GN, Lamarre AK, Treseler P. How I treat CNS lymphomas. Blood. 2013;122(14):2318-30.
19. Illerhaus G, Fritsch K, Egerer G, Lamprecht M, von Bubnoff N, Wolf HH, et al. Sequential High Dose Immuno-Chemotherapy Followed by Autologous Peripheral Blood Stem Cell Transplantation for Patients with Untreated Primary Central Nervous System Lymphoma - a Multicentre Study by the Collaborative PCNSL Study Group Freiburg. Blood. 2012;120(21).
20. Rubenstein JL, Hsi ED, Johnson JL, Jung SH, Nakashima MO, Grant B, et al. Intensive Chemotherapy and Immunotherapy in Patients With Newly Diagnosed Primary CNS Lymphoma: CALGB 50202 (Alliance 50202). J Clin Oncol. 2013.
21. Motomura K, Natsume A, Fujii M, Ito M, Momota H, Wakabayashi T. Long-term survival in patients with newly diagnosed primary central nervous system lymphoma treated with dexamethasone, etoposide, ifosfamide and carboplatin chemotherapy and whole-brain radiation therapy. Leuk Lymphoma. 2011;52(11):2069-75.
22. Takasu S, Wakabayashi T, Kajita Y, Hatano N, Hatano H, Usui T, et al. [Effectiveness of DeVIC chemotherapy for recurrent primary central nervous system lymphoma]. No Shinkei Geka - Neurological Surgery. 2000;28(9):789-94.
23. Correa DD, Maron L, Harder H, Klein M, Armstrong CL, Calabrese P, et al. Cognitive functions in primary central nervous

system lymphoma: literature review and assessment guidelines. Ann Oncol. 2007;18(7):1145-51.
24. Ferreri AJ, Blay JY, Reni M, Pasini F, Spina M, Ambrosetti A, et al. Prognostic scoring system for primary CNS lymphomas: the International Extranodal Lymphoma Study Group experience. Journal of Clinical Oncology. 2003;21(2):266-72.

---

*It is strongly recommended that this checklist be read in conjunction with the SPIRIT 2013 Explanation & Elaboration for important clarification on the items. Amendments to the protocol should be tracked and dated. The SPIRIT checklist is copyrighted by the SPIRIT Group under the Creative Commons "Attribution-NonCommercial-NoDerivs 3.0 Unported" license.
